# Polyamination with spermidine enhances pathogenic tau conformations while reducing filamentous aggregate formation *in vitro*

**DOI:** 10.1042/BCJ20253079

**Published:** 2025-06-17

**Authors:** Mohammed M. Alhadidy, Rebecca L. Mueller, Jared Lamp, Nicholas M. Kanaan

**Affiliations:** 1 Department of Translational Neuroscience, College of Human Medicine, Michigan State University, Grand Rapids, MI, U.S.A.; 2 Neuroscience Program, Michigan State University, East Lansing, MI, U.S.A.; 3 Current Affiliation: Structural Biology Department, Van Andel Institute, Grand Rapids, MI, U.S.A.; 4 Integrated Mass Spectrometry Unit, College of Human Medicine, Michigan State University, Grand Rapids, MI, U.S.A.; † Grand Rapids Research Center, 400 Monroe Ave NW, Grand Rapids, MI, USA

**Keywords:** Alzheimer’s disease, microtubules, oligomers, polyamination, post-translational modification, protein conformation, recombinant protein, tau, tauopathy

## Abstract

Tau is subject to a broad range of post-translational modifications (PTMs) that regulate its biological activity in health and disease, including microtubule (MT) dynamics, aggregation, and adoption of pathogenic conformations. The most studied PTMs of tau are phosphorylation and acetylation; however, the salience of other PTMs is not fully explored. Tissue transglutaminase (TG) is an enzyme whose activity is elevated in Alzheimer’s disease (AD). TG action on tau may lead to intramolecular and intermolecular cross-linking along with the incorporation of cationic polyamines (e.g., spermidine [SPD]) onto glutamine residues (Q). Even though SPD levels are significantly elevated in AD, the effects of SPD polyamination on tau biology have yet to be examined. In this work, we describe a method to produce recombinant SPD-modified tau where SPD modifications are mainly localized to Q residues within the N-terminus. MT binding and polymerization assays showed that SPD modification does not significantly alter tau’s binding to MTs but increases MT polymerization kinetics. In addition, biochemical and biophysical assays showed that SPD polyamination of tau markedly reduces tau polymerization into filamentous and β-sheet-containing aggregates. On the other hand, SPD modification promotes the formation of pathogenic conformations (e.g., oligomerization and misfolding) by tau with or without inducing tau polymerization. Taken together, these data suggest that SPD polyamination of tau enhances its ability to polymerize MTs and favors the adoption of pathogenic tau conformations but not filamentous aggregates *in vitro*.

## Introduction

Alzheimer’s disease (AD) and other tauopathies are characterized by the accumulation of abnormal forms of tau protein into intracellular inclusions that are closely associated with the progression of cognitive symptoms [1-9]. These disorders involve multiple cellular dysfunctions, including synaptic deficits, impaired axonal transport, mitochondrial impairment, and neuroinflammation, contributing to neurodegeneration [10-18]. Some of these cellular dysfunctions are related to tau’s interaction with the cytoskeleton, particularly its regulation of microtubule (MT) polymerization and dynamics [19,20]. However, the precise mechanisms by which tau contributes to cellular dysfunction in tauopathies continue to be actively investigated.

Under disease conditions, tau undergoes conformational alterations linked to neuronal dysfunction and degeneration, such as oligomerization [21-27], misfolding [28-30], and excessive exposure of the phosphatase-activating domain (PAD) [31-33]. Evidence suggests that these changes are regulated by tau mutations, protein–protein interactions, and post-translational modifications (PTMs) of tau [34].

PTMs, which occur across all domains of the tau protein, play a crucial role in regulating tau’s biology and pathobiology [34-37]. Tau PTMs modulate key cellular processes such as MT dynamics, proteasomal and autophagic clearance, and aggregation propensity [35,38-42]. Furthermore, disease-relevant PTMs influence the adoption of pathogenic conformations by tau, linking them to tauopathy mechanisms [35,39,43-45]. Phosphorylation and acetylation are the most extensively studied PTMs of tau, with several therapeutic strategies targeting these modifications under clinical investigation [34,46]. By comparison, PTMs such as SUMOylation, glycosylation, and polyamination remain underexplored, leaving significant gaps in our understanding of tau biology and its role in tauopathies [37,40,47-49].

Tissue transglutaminase (TG), an enzyme expressed in the central nervous system, is implicated in the development of AD and other tauopathies [50]. Elevated TG activity and expression in the prefrontal cortex of AD patients compared to controls suggest a potential role in disease pathology [51]. TG-catalyzed cross-linking was identified in tau purified from AD brains and P301L tau mouse models, further supporting this association [52,53]. TG catalyzes two types of reactions: cross-linking of glutamine (Q) and lysine (K) residues or the incorporation of polyamines (e.g., putrescine, spermidine [SPD], and spermine) onto Q residues [54-56]. Multiple studies confirm that tau is a substrate for TG catalytic activity [52,54-57]. The structural outcome of cross-linking, whether intramolecular or intermolecular, can influence whether tau forms misfolded filamentous structures [56,58]. While TG-catalyzed cross-linking is extensively studied, the potential consequences of polyamine incorporation onto Q residues in tau remain a nascent area of investigation.

Polyamines, including SPD, spermine, and putrescine, are ubiquitous polycationic amines in eukaryotic cells [59]. These molecules are essential for normal cellular functioning and survival [60,61]. Notably, SPD levels are significantly elevated in AD brains [62,63]. Research using tauopathy mouse models suggests that tau pathology disrupts polyamine metabolism, potentially exacerbating tau-related phenotypes through a feedforward mechanism [64-66]. Despite these findings, the role of SPD incorporation onto Q residues of tau currently represents a significant knowledge gap.

Although direct *in vivo* evidence linking SPD-mediated polyamination to tau pathology is limited, elevated SPD levels in AD brains and dysregulated polyamine pathways in tauopathy models suggest a potential biological role. Building on this premise, we generated recombinant SPD-modified 2N4R (SPD-hT40) and 2N3R (SPD-hT39) tau using TG-catalyzed *in vitro* polyamination reactions. Using biochemical and biophysical assays, we evaluated how SPD polyamination influences tau’s ability to bind MTs, affect MT polymerization, adopt pathogenic conformations, and aggregate *in vitro* [34]. Our results reveal that SPD polyamination of both tau isoforms enhances MT polymerization *in vitro*. Notably, SPD-modified hT40 shows an increase in pathogenic tau conformations and a reduction in filamentous tau structures during aggregation. Likewise, SPD modification of hT39 promotes the formation of larger globular aggregates and favors pathogenic conformations. These findings indicate that SPD polyamination alters tau’s regulation of MT dynamics, potentially contributing to tauopathies by promoting pathogenic conformations over filamentous aggregates.

## Results

### *In vitro* polyamination produces SPD-modified tau on several Q residues in multiple tau domains

**Figure 1:**
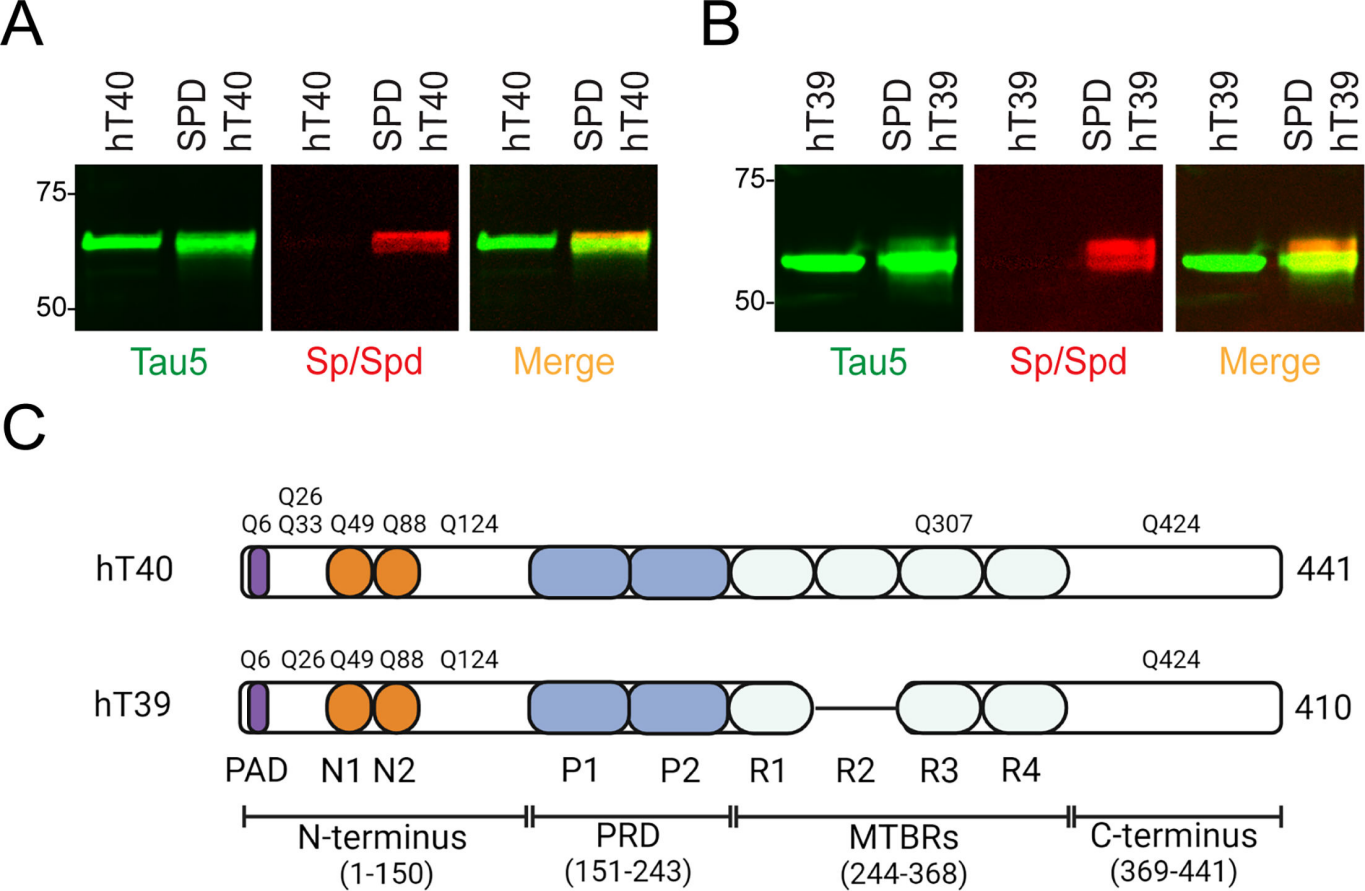
Identification of glutamine (**Q**) residues on tau modified with spermidine (SPD). (**A**) Western blot of unmodified 2N4R tau isoform (hT40) and SPD-modified hT40 (SPD-hT40) probed with the Tau5 antibody (green) and Spermine/SPD antibody (red). (**B**) Western blot of unmodified (hT39) and SPD-modified hT39 (SPD-hT39) probed with the Tau5 and Spermine antibodies. (**C**) Q residues modified with SPD on tau as detected by mass spectrometry. Residues are numbered according to the sequence of full-length tau (hT40, 441 amino acids). Q6 is located within the phosphatase-activating domain (PAD), a domain of tau whose abnormal exposure is linked to dysregulation of axonal transport. Q49 and Q88 are located within the N1 and N2 acidic inserts, respectively. Q307 was detected within the microtubule-binding repeat (MTBR) of SPD-hT40 but not SPD-hT39. Q424 is located within the C-terminus of both SPD-hT40 and SPD-hT39.

Western blotting with anti-tau and anti-SPD antibodies confirmed that the SPD-hT40 and SPD-hT39 proteins were polyaminated with SPD (Figure 1A and B). The unmodified hT40 and hT39 controls did not show SPD signal, while all tau proteins were labeled with Tau5 (Figure 1A and B).

Next, MS was used to provide additional insight into the specific Q residues modified by SPD in hT40 and hT39 proteins. MS results confirm that both SPD-hT40 and SPD-hT39 were polyaminated with SPD at several residues. Both proteins share similar modified Q residues throughout the tau protein, including residues Q6, Q26, Q49, Q88, Q124, and Q424 (Figure 1C). SPD modifications on Q33 and Q307 were detected in SPD hT40 but not SPD hT39 (Figure 1C). Notably, the majority of modifications were in the N-terminus of both tau isoforms. Sample mass spectra (MS) of modified (from SPD-hT40 or SPD-hT39 samples) and unmodified (from hT40 or hT39 samples) versions of the tau peptides spanning amino acids 407–438 are provided as representative examples in Supplementary Figure S2 and S3. It is noteworthy that the unmodified hT40 and hT39 control proteins did not show MS-based evidence of SPD modifications. Processed proteomics data on tau peptides are available in this manuscript (Supplementary Tables S1-S3 and Supporting Information), and full proteomics data sets and .RAW files from MS are available in a public repository (http://datadryad.org/stash/share/sZqDX8ucGqxJJT4nvuVJisk0Sf71OABbLx8hxwec9tw).

### SPD-modified tau accelerates MT polymerization *in vitro*

**Figure 2:**
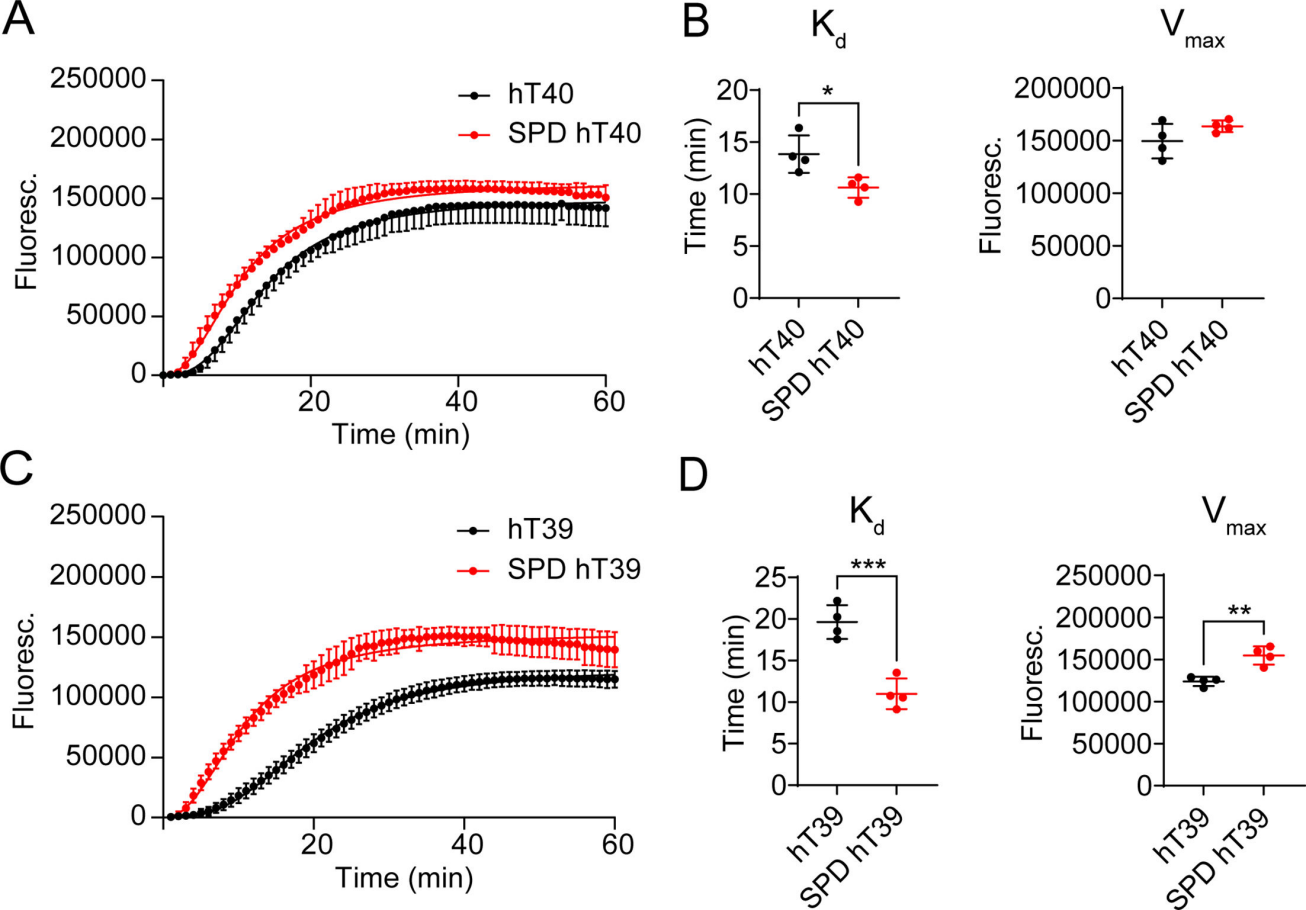
Polyamination of tau with spermidine (SPD) accelerates tubulin polymerization *in vitro*. (**A**) Curve fit of fluorescence signal corresponding to microtubule (MT) polymerization in the presence of either unmodified 2N4R tau isoform (hT40; black) or SPD-modified hT40 (SPD-hT40; red) proteins over 60 minutes. (**B**) Dissociation constant (*Kd*) of MT polymerization for unmodified hT40 and SPD-hT40 proteins (left panel). Maximum velocity (*V*_max_) of MT polymerization for unmodified hT40 and SPD-hT40 (right panel). (**C**) Curve fit of fluorescence signal corresponding to MT polymerization in the presence of either unmodified hT39 (black) or SPD-hT39 (red) proteins over 60 minutes. (**D**) *Kd* of MT polymerization for unmodified hT39 and SPD-hT39 proteins (left panel). *V*_max_ of MT polymerization for unmodified hT39 and SPD-hT39 (right panel). Data represented as mean ± standard deviation (SD) from four independent sets of samples (*n* = 4). *P≤0.05; **P≤0.01; ***P≤0.001; ****P≤0.0001.

Tubulin polymerization assays were used to determine how SPD modification affects the ability of unmodified and SPD-modified hT40 (Figure 2A) or hT39 (Figure 2C) to modulate MT polymerization kinetics *in vitro*. SPD-hT40 exhibited significantly decreased time to half-maximal polymerization (Figure 2B; *Kd*, *t* = 3.115, *P*<0.05), with no change in the steady state equilibrium (Figure 2B; *V*_max_, *t* = 1.675, *P*>0.05) when compared with hT40. SPD-hT39 exhibited significantly decreased time to half-maximal polymerization (Figure 2D; *Kd*, *t* = 6.977, *P*<0.05) and increased steady state equilibrium (Figure 2D; *V*_max_, *t* = 5.614, *P*<0.05) when compared with hT39.

### SPD modification of tau alters its ability to bind MTs *in vitro*

**Figure 3:**
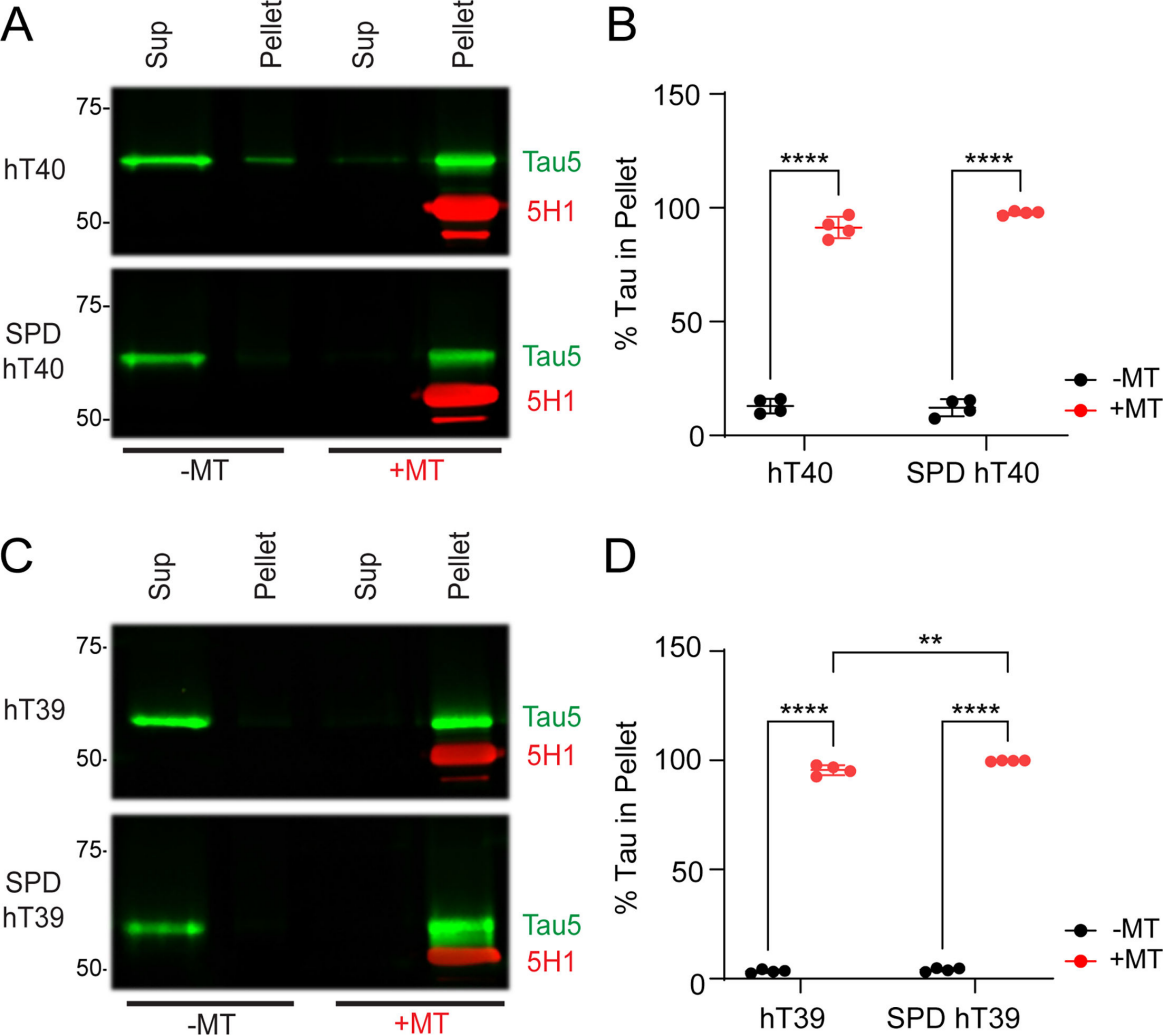
Polyamination of tau with spermidine (SPD) does not robustly alter binding of tau to microtubules (MTs) *in vitro*. (**A**) Representative western blot of MT-binding assay for unmodified 2N4R tau isoform (hT40) and SPD-modified hT40 (SPD-hT40) proteins using Tau5 and 5H1 (tubulin) antibodies. (**B**) Quantification of the fraction of tau detected in the pellet of polymerized MTs for hT40 and SPD-hT40. (**C**) Representative western blot of MT-binding assay for unmodified 2N3R tau isoform (hT39) and SPD-hT39 proteins using Tau5 and 5H1 antibodies. (**D**) Quantification of the fraction of tau detected in the pellet of polymerized MTs for hT39 and SPD-hT39. Note that while the difference between hT39 and SPD-hT39 + MT was statistically significant, the difference was only a 4.5% increase in SPD-hT39 in the pellet. Data represented as mean ± SD from four independent sets of samples (*n* = 4). *P≤0.05; **P≤0.01; ***P≤0.001; ****P≤0.0001.

MT-binding assays were used to determine how SPD modification affects tau’s ability to bind preformed MTs. Experiments were conducted using either the hT40 or hT39 tau isoforms independently (Figure 3A and C). Two-way ANOVA revealed a significant main effect for tau in the MT fraction (*F*_(1,12)_ = 2300, *P*<0.05). Regardless of SPD modification status, post-hoc testing indicated a statistically significant increase in % of tau in the pellet when compared with the supernatant for hT40 and SPD-hT40 proteins (Figure 3B; *t* = 32.44, *P*<0.05 for hT40; *t* = 35.37, *P*<0.05 for SPD-hT40).

Two-way ANOVA detected a statistically significant interaction between the PTM status of hT39 proteins and the MT fraction (*F*_(1,12)_ = 7.304, *P*<0.05). Regardless of SPD modification status, there was a significant increase in % of tau in the pellet when compared with the supernatant for hT39 and SPD-hT39 proteins (Figure 3D; *t* = 100.1, *P*<0.05 for hT39; *t* = 103.9, *P*<0.05 for SPD hT39). Furthermore, the % of SPD-hT39 in the MT pellet fraction showed a slight (4.5%) but significant increase when compared with that of hT39 (Figure 3D; *t* = 4.609, *P*<0.05).

### SPD modification of tau alters its aggregation kinetics *in vitro*

**Figure 4:**
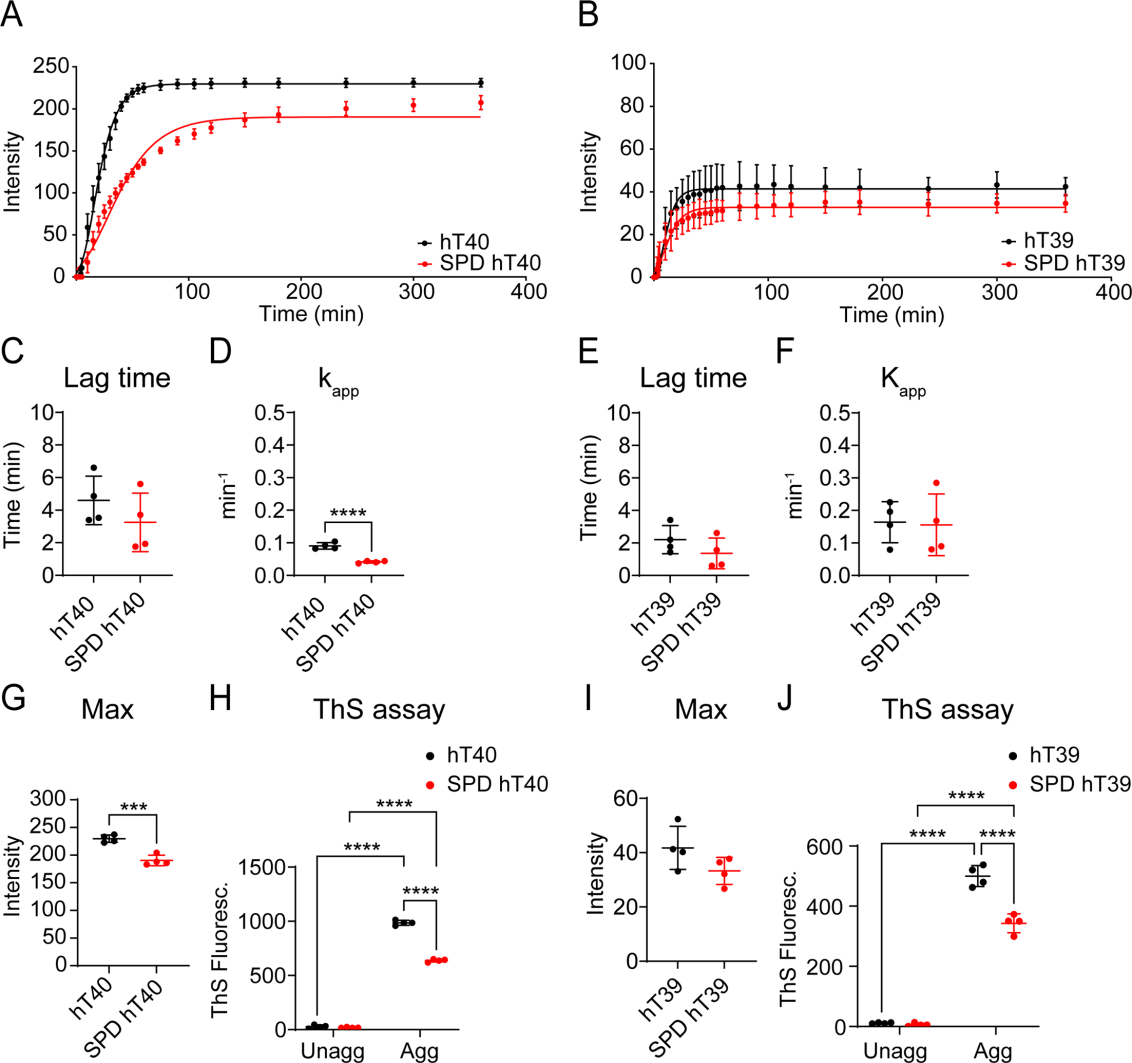
Spermidine (SPD) modification decreases the rate and extent of tau multimerization, while reducing β-sheet-containing aggregates *in vitro*. (**A**) Laser light scattering (LLS) intensity of unaggregated (unagg) and aggregated (agg) 2N4R tau isoform (hT40) and SPD-modified hT40 (SPD-hT40) proteins. (**B**) LLS intensity of unagg and agg 2N3R tau isoform (hT39) and SPD-hT39 proteins. (**C**) Lag time to aggregation for hT40 and SPD-hT40 proteins. (**D**) Rate of tau multimerization (*k*_app_) for hT40 and SPD-hT40 proteins. (**E**) Lag time to aggregation for hT39 and SPD-hT39 proteins. (**F**) *k*_app_ for hT39 and SPD-hT39 proteins. (**G**) Maximum light scattering (max) polymerization for hT40 and SPD-hT40 proteins. (**H**) Thioflavin S (ThS) fluorescence signal after 6 hours of tau aggregation for hT40 and SPD-hT40 proteins. (**I**) Max polymerization for hT39 and SPD-hT39 proteins. (**J**) ThS fluorescence signal after 6 hours of tau aggregation for hT39 and SPD-hT39 proteins. Data represented as mean ± SD from four independent sets of samples (*n* = 4). *P≤0.05; **P≤0.01; ***P≤0.001; ****P≤0.0001.

Right-angle laser light scattering (LLS) assays were used to determine the impact of SPD modification on the kinetics of tau aggregation *in vitro* (Figure 4A and B). We observed an 18% reduction in the maximum light scattering for SPD-hT40 when compared with hT40 (Figure 4G; *t* = 6.796, *P*<0.05). Moreover, SPD modification slowed down the aggregation rate of hT40 by 45% (Figure 4D; *t* = 9.276, *P*<0.05), with no difference in lag time (Figure 4C; *t* = 1.152, *P*>0.05). On the other hand, SPD modification did not significantly change the aggregation kinetics of hT39 protein (Figure 4E, F and I; *t* = 1.152, *P*>0.05 for lag time; *t* = 0.1427, *P*>0.05 for *k*_app_; *t* = 1.814, *P*>0.05 for max).

### SPD modification of tau reduces β-sheet-containing aggregates *in vitro*

Thioflavin S (ThS) assays were performed at the end of aggregation reactions to determine the extent of β-sheet-containing aggregate formation *in vitro*. Two-way ANOVA detected a statistically significant interaction between the PTM and aggregation of hT40 (*F*_(1, 12)_ = 480.7, *P*<0.05) and hT39 (*F*_(1,12)_ = 41.56, *P*<0.05) proteins. Upon aggregating hT40 and hT39 proteins, there was a significant increase in the ThS signal compared to their respective unaggregated samples regardless of the SPD modification (Figure 4H and J; *t* = 87.64, *P*<0.05 for hT40; *t* = 56.63, *P*<0.05 for SPD-hT40; *t* = 29.37, *P*<0.05 for hT39; *t* = 20.25, *P*<0.05 for SPD-hT39). Of note, the SPD-modified aggregates of tau had significantly less ThS signal compared with the unmodified aggregated tau proteins (Figure 4H and J; *t* = 31.85 for hT40, *P*<0.05; *t* = 9.433 for hT39, *P*<0.05).

### SPD modification alters the formation of tau aggregates *in vitro*

**Figure 5:**
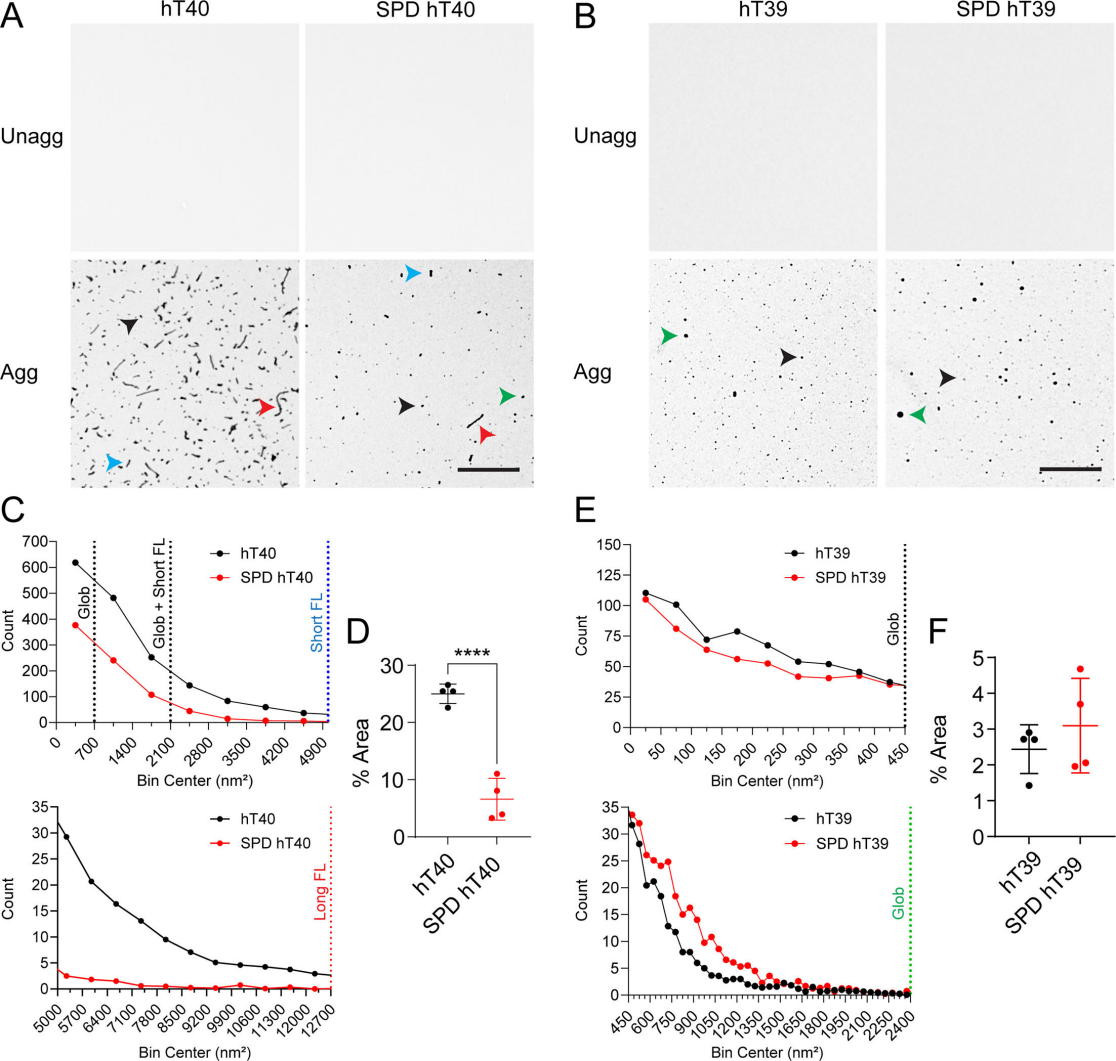
Spermidine (SPD) modification alters the size distribution of tau aggregates *in vitro*. (**A**) Electron micrographs of unaggregated (unagg) and aggregated (agg) 2N4R tau isoform (hT40) or SPD-modified hT40 (SPD-hT40) proteins. Scale bar: 800 nm. Globular aggregates <700 nm² are highlighted with black arrowheads; short (2100–5000 nm²) and long (>5000 nm²) filaments are highlighted with blue and red arrowheads, respectively. (**B**) Electron micrographs of unagg and agg 2N3R tau isoform (hT39) or SPD-hT39 proteins. Scale bar: 800 nm. Globular aggregates <450 nm² are highlighted with black arrowheads; globular aggregates > 450 nm² are highlighted with green arrowhead. (**C**) Size distribution of aggregates formed by hT40 and SPD-hT40 proteins. (**D**) Total mass of aggregates observed with hT40 and SPD-hT40 expressed as percentage of field area. (**E**) Size distribution of aggregates formed by hT39 and SPD-hT39 proteins. (**F**) Total mass of aggregates observed with hT39 and SPD-hT39 expressed as percentage of field area. Data represented as mean ± SD from four independent sets of samples (*n* = 4). *P≤0.05; **P≤0.01; ***P≤0.001; ****P≤0.0001.

Unaggregated and aggregated tau samples were imaged using TEM to assess morphology and quantify aggregate sizes. There was a 74% reduction in the total mass (% area) of aggregates for SPD-hT40 when compared with hT40 (Figure 5A and D; *t* = 9.163, *P*<0.05). Furthermore, the number of globular ( < 700 nm^2^), short filamentous (2100–5000 nm^2^), and long filamentous ( > 5000 nm^2^) aggregates was reduced with SPD modification of hT40 (Figure 5C, upper and lower panels).

No difference was observed in the aggregated mass (% area) between hT39 and SPD-hT39 samples (Figure 5B and F; Mann–Whitney *U* = 6, *P*>0.05). The size distribution of aggregates showed that SPD-hT39 proteins form less globular aggregates <450 nm^2^ along with a higher number of globular aggregates >450 nm^2^ (Figure 5E, upper and lower panels).

### SPD modification increases the stable multimers in unaggregated and aggregated tau *in vitro*

**Figure 6:**
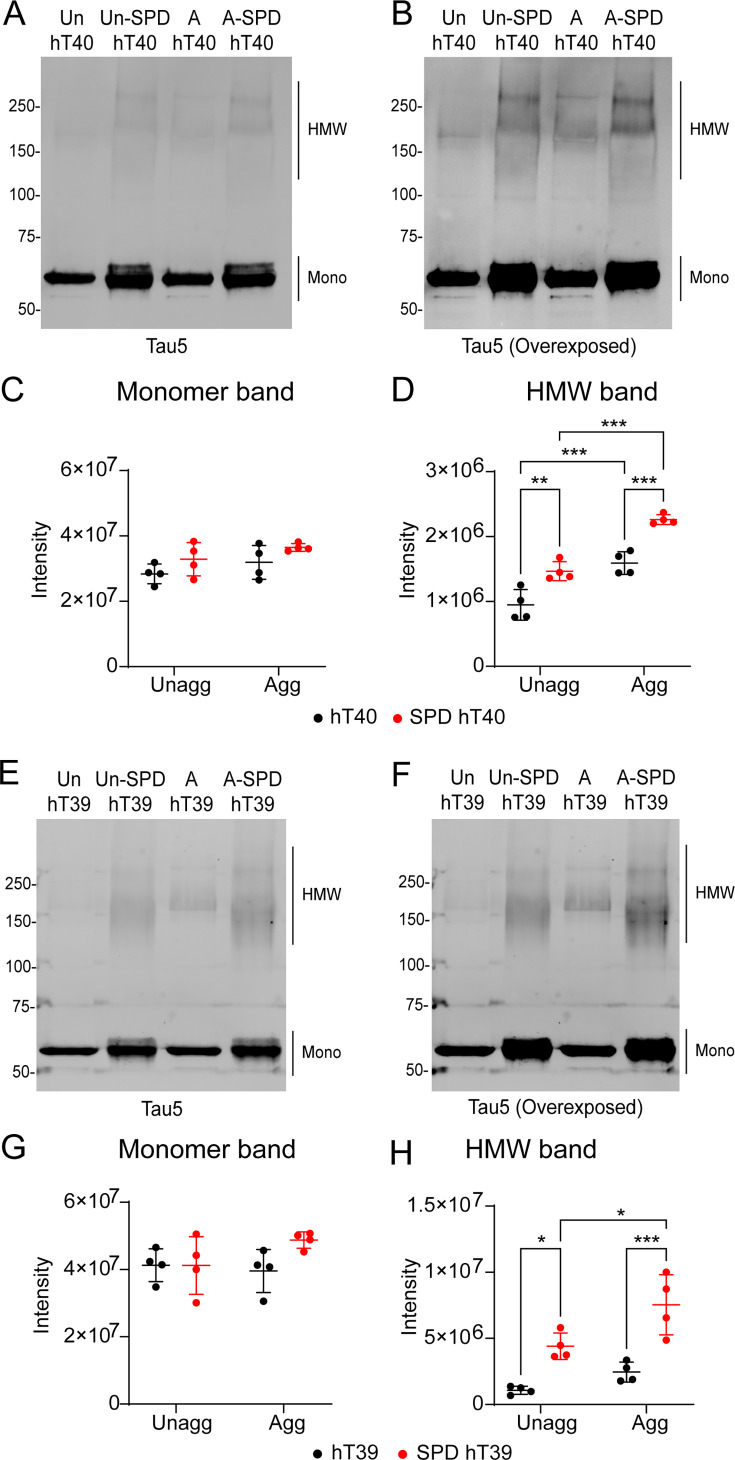
Spermidine (SPD) modification increases stable multimers in unaggregated (unagg) and aggregated (agg) tau samples. (**A-B**) Western blot of unagg and agg samples of unmodified 2N4R tau isoform (hT40) and SPD-modified hT40 (SPD-hT40) samples probed with Tau5 antibody (**A**) and an enhanced exposure to highlight the HMW bands (**B**). (**C**) Quantification of monomeric tau bands in hT40 and SPD-hT40 samples. (**D**) Quantification of the high molecular weight (HMW) stable multimer tau bands in hT40 and SPD-hT40 samples. (**E-F**) Western blot of unagg and agg samples of unmodified 2N3R tau isoform (hT39) and SPD-hT39 samples probed with Tau5 antibody (**E**) and an enhanced exposure to highlight the HMW bands (**F**). (**G**) Quantification of monomeric tau bands in hT39 and SPD-hT39 samples. (**H**) Quantification of HMW tau bands in hT39 and SPD-hT39 samples. Data represented as mean ± SD from four independent sets of samples (*n* = 4). *P≤0.05; **P≤0.01; ***P≤0.001; ****P≤0.0001.

Tau aggregation leads to the formation of heat-, SDS-, and reducing condition-stable multimers. Here, SDS-PAGE and western blotting were used to assess the extent to which SPD modification of tau affects the formation of these stable multimers (Figure 6A, B, E and F). A significant increase in the stable multimers (high molecular weight bands) upon aggregating was observed in hT40 and SPD-hT40 samples (Figure 6D; two-way ANOVA, Aggregation factor: *F*_(1, 12)_ = 72.79, *P*<0.05; *t* = 5.401, *P*<0.05 for unmodified hT40; *t* = 6.665, *P*<0.05 for SPD-hT40). Moreover, stable multimers were 40% higher in SPD-hT40 compared to hT40 (Figure 6D; two-way ANOVA, PTM factor: *F*_(1, 12)_ = 49.70, *P*<0.05; *t* = 5.617, *P*<0.05). Notably, stable multimers were 50% higher in the unaggregated SPD-hT40 relative to the unaggregated unmodified hT40 (Figure 6D; *t* = 4.353, *P*<0.05). Even though we detected a significant main effect of PTM on the monomeric tau signal (Figure 6C; *F*_(1, 12)_ = 5.158, *P*<0.05), post-hoc testing did not reveal any statistically significant differences between the hT40 and SPD-hT40 proteins.

Stable multimers (high molecular weight bands) were significantly elevated upon aggregating SPD-hT39 but not hT39 proteins (Figure 6H; two-way ANOVA, Aggregation factor: *F*_(1, 12)_ = 11.89, *P*<0.05; *t* = 1.492, *P*>0.05 for unmodified hT39; *t* = 3.385, *P*<0.05 for SPD-hT39). Furthermore, SPD-hT39 had higher levels of stable multimers in both the unaggregated and aggregated samples when compared to hT39 (Figure 6H; two-way ANOVA, PTM factor: *F*_(1, 12)_ = 41.55, *P*<0.05; *t* = 3.611, *P*<0.05 for unaggregated samples; *t* = 5.505, *P*<0.05 for aggregated samples). No significant differences were observed in the monomeric tau signal in the unaggregated and aggregated hT39 or SPD-hT39 proteins (Figure 6G).

### SPD modification of tau enhances the formation of pathogenic conformations *in vitro*

**Figure 7:**
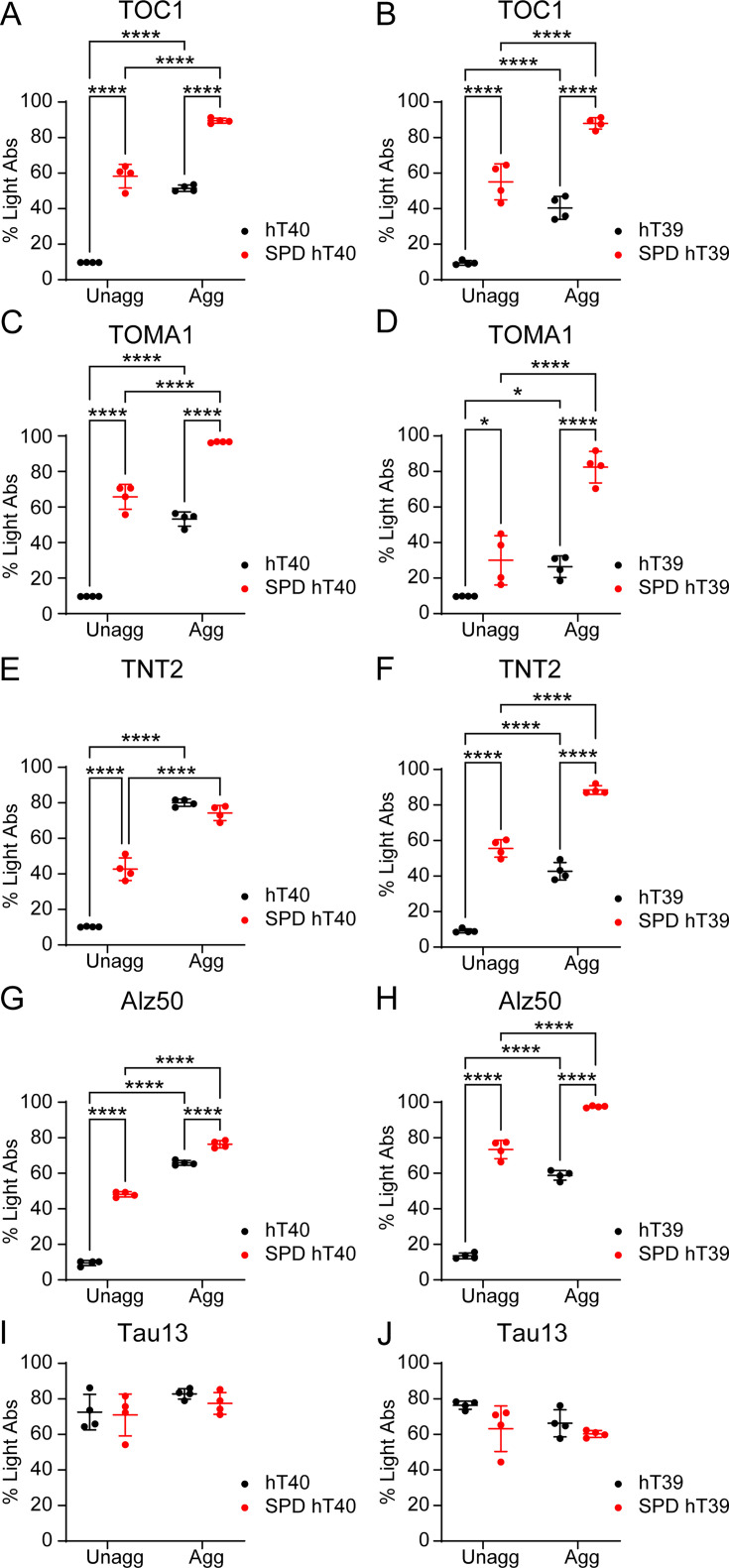
Spermidine (SPD) modification of tau increases the formation of pathogenic conformations independent of aggregation *in vitro*. (**A-B**) sELISAs measuring TOC1-positive oligomeric tau in unaggregated (unagg) and aggregated (agg) samples of unmodified 2N4R tau isoform (hT40) and SPD-modified hT40 (SPD-hT40) (**A**) or 2N3R tau isoform (hT39) and SPD-hT39 (**B**) proteins. The TOC1 antibody was used for capture and R1 antibody for detection. (**C-D**) sELISAs measuring TOMA1-positive oligomeric tau in unagg and agg hT40 and SPD-hT40 (**C**) or hT39 and SPD-hT39 (**D**) proteins. The TOMA1 antibody was used for capture and R1 antibody for detection. (**E-F**) sELISAs measuring PAD exposure in unagg and agg hT40 and SPD-hT40 (**E**) or hT39 and SPD-hT39 (**F**) proteins. The TNT2 antibody was used for capture and R1 antibody for detection. (**G-H**) sELISAs measuring Alz50-positive misfolded tau conformation in unagg and agg hT40 and SPD-hT40 (**G**) or hT39 and SPD-hT39 (**H**) proteins. The Alz50 antibody was used for capture and R1 antibody for detection. (**I-J**) sELISA measuring total tau in unagg and agg hT40 and SPD-hT40 (**I**) or hT39 and SPD-hT39 (**J**) proteins using Tau13 antibody for capture and R1 antibody for detection. Data represented as mean percentage of light absorbed (%Light Abs) ± SD from four independent sets of samples (*n* = 4). *P≤0.05; **P≤0.01; ***P≤0.001; ****P≤0.0001.

Several antibodies are available to detect oligomeric tau species, which are linked to toxicity and neurodegeneration [21,34,67,68]. Oligomeric tau was quantified in unaggregated and aggregated tau samples using sandwich enzyme-linked immunosorbent assays (sELISAs) with two different oligomeric tau antibodies (i.e., TOC1 and TOMA1). The assays showed an increase in TOC1-positive (Figure 7A; two-way ANOVA, Interaction: *F*_(1, 12)_ = 8.959, *P*<0.05; *t* = 16.75, *P*<0.05 for hT40; *t* = 12.51, *P*<0.05 for SPD hT40) and TOMA1-positive (Figure 7C; two-way ANOVA, Interaction: *F*_(1, 12)_ = 9.751, *P*<0.05; *t* = 15.23, *P*<0.05 for hT40; *t* = 10.82, *P*<0.05 for SPD-hT40) oligomeric tau in the aggregated samples of hT40 and SPD-hT40 relative to their respective unaggregated proteins. Of note, the unaggregated SPD-hT40 samples had significantly higher levels of both oligomeric tau species relative to hT40 (Figure 7A, *t* = 19.48, *P*<0.05 for TOC1; Figure 7C, *t* = 19.60, *P*<0.05 for TOMA1). Moreover, the aggregated samples of SPD-hT40 contained higher levels of oligomeric tau relative to the aggregated hT40 (Figure 7A, *t* = 15.24, *P*<0.05 for TOC1; Figure 7C, *t* = 15.19, *P*<0.05 for TOMA1).

The patterns observed with hT40 proteins were the same for hT39 proteins (Figure 7B and D). Increases in TOC1-positive (Figure 7B, two-way ANOVA, Aggregation factor: *F*_(1, 12)_ = 104.2, *P*<0.05; *t* = 6.99, *P*<0.05 for hT39; *t* = 7.444, *P*<0.05 for SPD-hT39) and TOMA1-positive (Figure 7D, two-way ANOVA, Interaction: *F*_(1, 12)_ = 16.57, *P*<0.05; *t* = 2.668, *P*<0.05 for hT39; *t* = 8.426, *P*<0.05 for SPD-hT39) oligomeric tau species were detected in the aggregated samples of hT39 and SPD-hT39 relative to their respective unaggregated proteins. Notably, the unaggregated SPD-hT39 samples had significantly higher levels of both oligomeric tau species relative to hT39 (Figure 7B, two-way ANOVA, PTM factor: *F*_(1, 12)_ = 221.9, *P*<0.05 for TOC1; *t* = 10.31, *P*<0.05 for TOC1; Figure 7D, *t* = 3.25, *P*<0.05 for TOMA1). Moreover, the aggregated samples of SPD-hT39 contained higher levels of oligomeric tau relative to the aggregated hT39 (Figure 7B, *t* = 10.76, *P*<0.05 for TOC1; Figure 7D, *t* = 9.007, *P*<0.05 for TOMA1).

Tau adopts pathogenic conformations associated with modifications of monomeric tau and following multimerization that are also linked to toxicity [31,34,44,69]. Those conformations include exposure of the N-terminal PAD (amino acids 2–18), which is linked to dysfunction of axonal transport [31,70-74]. The extent to which SPD modification can influence the adoption of a PAD-exposed conformation was assessed using sELISAs with the TNT2 antibody. Aggregated samples showed significantly higher TNT2 signal compared with their unaggregated counterparts (Figure 7E, two-way ANOVA, Interaction: *F*_(1, 12)_ = 93.47, *P*<0.05) in the hT40 samples (*t* = 25.01, *P*<0.05) and the SPD-hT40 samples (*t* = 11.34, *P*<0.05). Moreover, TNT2 signal was significantly higher in unaggregated SPD-modified hT40 relative to unmodified hT40 (Figure 7E, *t* = 11.61, *P*<0.05), suggesting this modification may expose PAD without aggregation. There was not a significant difference in TNT2 signal between aggregated hT40 and aggregated SPD-hT40 (*t* = 2.059, *P*>0.05).

We also assessed the impact of SPD modification on PAD exposure of the hT39 isoform. Aggregated samples showed significantly higher TNT2 signal compared to their unaggregated counterparts (Figure 7F, two-way ANOVA, Aggregation factor: *F*_(1, 12)_ = 316.7, *P*<0.05) in the hT39 samples (*t* = 12.70, *P*<0.05) and the SPD-hT39 samples (*t* = 12.47, *P*<0.05). Moreover, TNT2 signal was significantly higher in unaggregated SPD-hT39 relative to hT39 (Figure 7F, two-way ANOVA, PTM factor: *F*_(1, 12)_ = 609.2, *P*<0.05; *t* = 17.57, *P*<0.05), suggesting this modification may expose PAD without aggregation. There was also a significant difference in TNT2 signal between aggregated hT39 and aggregated SPD-hT39 (*t* = 17.34, *P*<0.05).

Another misfolded conformation involves the N-terminus of tau coming into close proximity with the MT-binding region (detectable with the conformation-specific Alz50 antibody), and it represents an aggregation-prone folding event that occurs early in disease [29,30,75]. The extent to which SPD modification can influence the adoption of Alz50 conformation was assessed using sELISAs. Alz50-positive tau was significantly higher in the aggregated samples relative to their respective unaggregated proteins (Figure 7G, two-way ANOVA, Interaction: *F*_(1, 12)_ = 313.1, *P*<0.05) in the hT40 tau samples (*t* = 50.08, *P*<0.05) and SPD-hT40 samples (*t* = 25.05, *P*<0.05). Moreover, the Alz50-positive conformation was more abundant in unaggregated SPD-hT40 relative to hT40 (Figure 7G, *t* = 34.33, *P*<0.05). Unlike TNT2 signal, aggregated SPD-hT40 showed higher Alz50-positive tau relative to hT40 aggregates (*t* = 9.310, *P*<0.05).

We also assessed the degree of tau misfolding in unaggregated and aggregated hT39 samples. Aggregated samples showed significantly higher Alz50 signal compared with their unaggregated counterparts (Figure 7H, two-way ANOVA, Interaction: *F*_(1, 12)_ = 48.87, *P*<0.05) in the hT39 samples (*t* = 21.11, *P*<0.05) and the SPD-hT39 samples (*t* = 11.23, *P*<0.05). Moreover, Alz50 signal was significantly higher in unaggregated SPD-hT39 relative to hT39 (Figure 7H, *t* = 27.91, *P*<0.05), suggesting this modification may induce tau misfolding without aggregation. There was also a significant increase in Alz50 signal in the aggregated SPD-hT39 relative to the aggregated hT39 (*t* = 18.02, *P*<0.05).

For all unaggregated and aggregated hT40 and hT39 proteins, total tau levels were measured using Tau13 as a capture antibody (Figure 7I and J). No significant differences were observed in total tau levels within the hT40 samples (Figure 7I). Even though we detected a significant main effect of PTM on total tau signal for hT39 samples (two-way ANOVA, *F*_(1, 12)_ = 6.355, *P*<0.05), post-hoc testing did not reveal any statistically significant differences between hT39 and SPD-hT39 samples in unaggregated and aggregated states (Figure 7J).

### Discussion

Tau undergoes numerous PTMs under both normal and pathological conditions, with phosphorylation and acetylation being the most extensively studied [34]. These modifications regulate essential aspects of tau biology, including its interactions with other proteins, aggregation behavior, conformational plasticity, MT binding, and mechanisms of degradation and clearance [34,37]. However, the effects of other PTMs, such as TG-mediated polyamination, remain largely unexplored [52,54-57]. Early studies primarily examined TG-mediated cross-linking of tau, with little attention given to polyamine incorporation onto glutamine residues [55]. Interestingly, metabolic profiling of polyamine levels, which may be linked to TG-mediated reactions, indicated increased SPD levels in AD brains [62,63]. Furthermore, studies in animal models of tauopathy reported a polyamine stress response triggered by tau pathology that may exacerbate its progression [64-66]. Thus, understanding the consequences of SPD incorporation onto glutamine residues is critical for elucidating its role in tau pathology [62,63].

This study was designed to fill a critical gap in understanding TG-mediated polyamination and its effects on tau biology. We generated SPD-modified tau to explore its influence on MT binding, MT polymerization, the adoption of pathogenic conformations, and aggregation behavior *in vitro*. TG-catalyzed reactions with SPD resulted in the polyamination of multiple glutamine residues, predominantly within the N-terminus of tau. The modifications were observed in both hT40 and hT39 tau isoforms at Q6, Q26, Q49, Q88, Q124, and Q424, while Q33 and Q307 were unique to hT40. Notably, Q307 and Q424 were previously identified as amine acceptors in TG-mediated cross-linking, highlighting their importance in tau biology [54]. Furthermore, Q6, Q88, and Q124 were shown to act as amine acceptors in TG-mediated cross-linking of hT40 [54].

Tau’s well-established role in regulating MT dynamics [76-81] prompted us to investigate how SPD modification affects its binding and polymerization properties. While SPD modification did not significantly alter the binding of hT40 or hT39 tau isoforms to preformed MTs, it markedly accelerated MT polymerization rates. Additionally, SPD-hT39 enhanced the overall extent of polymerization, suggesting that polyamination modulates tau’s regulatory functions on MT dynamics. Given that polyamines carry positive charges [59] and MTs are negatively charged [82], electrostatic interactions between SPD-tau and tubulin likely contribute to the enhanced polymerization observed in our study. Another explanation for the accelerated MT polymerization could be attributed to the localization of SPD modification mainly within the N-terminus of tau. Recently, the Rhoades group showed that the N-terminus of tau negatively regulates tubulin’s interactions with the proline-rich domain and the microtubule-binding repeat region of tau [83]. Thus, incorporating SPD into the N-terminus of tau may mitigate these inhibitory effects, enhancing interactions between tubulin and tau. The data suggest that polyamination of tau may have implications in the physiological and potentially disease-associated roles of tau related to MT binding and regulation, warranting further investigation.

PTMs are regulators of tau biology and are intimately linked to processes associated with tau in human disease, including the adoption of pathogenic conformations in monomeric or multimeric tau species [34]. Sustained conformation-dependent exposure of PAD is a pathological event that occurs early in AD and is linked to dysregulation of axonal transport [31]. This dysregulation is mediated by the activation of a signaling pathway involving protein phosphatase 1 and glycogen synthase kinase 3β, triggered by PAD exposure [31,44,70,71]. In this study, we showed that SPD modification of tau induces conformational changes that increase PAD exposure in both aggregated and unaggregated tau species. These findings suggest that SPD modification enhances PAD exposure, potentially contributing to axonal transport impairment independent of tau aggregation status. Interestingly, the effects of SPD polyamination on PAD may resemble those observed with other specific PTMs and mutant forms of tau, such as phosphomimics at sites within the AT8 epitope and the P301L mutation [31,44,69,71,84].

Oligomerization of tau occurs in early disease stages before the development of cognitive symptoms in AD patients [22,27]. Substantial evidence indicates that oligomeric tau species mediate dysfunction and degeneration, challenging the traditional view of tau filaments as the primary toxic entities [85]. Indeed, tau oligomers are linked to several disease mechanisms including axonal transport impairment [24,31,70-74,86], mitochondrial and synaptic dysfunction [87], reduced protein synthesis [88], inhibited long-term potentiation [89], and memory impairment [90]. Notably, immunotherapies targeting oligomeric tau species reverse the tauopathy phenotype in rodent models, highlighting the central role of tau oligomers in disease progression [68,91]. SPD modification of tau significantly increases the formation of oligomeric tau species under aggregation-inducing conditions. Furthermore, it promoted oligomer formation even in unaggregated tau samples (i.e., without the ARA inducer). These findings suggest that polyamination may facilitate oligomer formation and exacerbate the toxic mechanisms of tau oligomers, which are known to play a critical role in tauopathy progression.

Tau also assumes a misfolded conformation in disease conditions where the N-terminus folds onto the MT-binding region, and it is thought to precede the formation of filamentous tau aggregates [30]. This misfolded conformation is detected using the Alz50 antibody, originally developed to target paired helical filaments isolated from AD brain tissue [92]. SPD modification of tau was found to promote the Alz50-positive pathogenic conformation in both aggregated and unaggregated samples. These findings are consistent with prior studies demonstrating that PTMs drive the adoption of Alz50-like conformations, underscoring polyamination as another potential regulator of tau misfolding [93].

SPD modification decreases the extent of filamentous tau aggregation, as indicated by reduced β-sheet structure formation (ThS) and LLS signal. Previous studies demonstrated that removing the N-terminus of tau diminishes its *in vitro* aggregation, suggesting that the N-terminus plays a key role in facilitating filamentous aggregation [94]. Therefore, it is plausible that preferential SPD modification in the N-terminus disrupts its pro-aggregation role, thereby inhibiting filamentous aggregation. In agreement with the overall reduction in aggregation, TEM demonstrated that SPD polyamination of tau markedly reduces the formation of both globular and filamentous tau aggregates. Conversely, SPD modification also significantly increased levels of oligomeric, PAD-exposed, and Alz50-positive tau conformations, even in the absence of aggregation-inducing conditions. The observed pattern of tau polymerization following SPD polyamination supports the notion that pathogenic tau conformations are not inherently tied to filamentous aggregate formation. These findings suggest that tau polymerization can follow either an on-filament pathway, leading to filamentous aggregates, or an off-filament pathway, resulting in non-filamentous species [95]. Supporting this view, we also found that 4R SPD-tau did not seed aggregation in a cell seeding assay using the TauRD biosensor cell line [96], but 3R SPD-tau seeding was not tested.

Taken together, our data suggest that SPD polyamination promotes off-filament pathway tau polymerization. The observation that SPD polyamination enhances pathogenic conformations while reducing filamentous aggregation is potentially significant given emerging evidence that tau oligomers and/or modified monomers, rather than filaments, are likely the primary drivers of neurotoxicity in tauopathies. This study not only advances our understanding of tau PTMs but also suggests a mechanism by which elevated SPD levels may contribute to disease progression.

In the context of AD, TG activity and SPD levels are significantly elevated, indicating that polyamination pathways may be dysregulated in the disease [51,62,63]. Putrescine and spermine, polyamines that may also serve as potential tau-modifying PTMs, are also dysregulated in AD and tauopathies [62,63]. Prior work demonstrated that free SPD, spermine, putrescine, and their acetylated derivatives have differential effects on heparin-induced filamentous tau aggregate formation *in vitro* [64,65]. Aligned with our data, these studies showed that free SPD reduces filamentous tau aggregation (as assayed using ThT assays) and seeding (in cell-based aggregation assays). These findings highlight the importance of continuing investigations into the impacts of free polyamines and modification of tau with polyamines on the biological and pathobiological roles of tau.

Modulating polyamine metabolism significantly affects tauopathy phenotypes in mouse models. Using an adeno-associated virus (AAV) C-terminal truncated tau mouse model, the Lee group showed that knocking out the SPD/spermine-N1-acetyltransferase enzyme (SSAT) decreases putrescine and SPD levels, reduces loss of neurons in the hippocampus and cortex (NeuN+neurons), but does not change the levels of insoluble tau aggregates [65]. Conversely, AAV-mediated overexpression of arginase 1, a polyamine metabolism enzyme, in the rTg4510 mouse model reduced tau tangle-like pathology in the hippocampus (i.e., Gallyas silver-positive inclusions) and formic acid insoluble tau, although it did not prevent overt neuron loss [66]. These studies suggest that polyamine pathways are involved in tau-mediated toxicity likely through non-filamentous pathogenic tau species. This observation aligns with our findings that SPD modification of tau favors the formation of pathogenic tau conformations over filamentous aggregates. It is noteworthy that the level of tau polyamination modification was not directly examined in the above *in vivo* studies, highlighting the need for further research to clarify the role of tau polyamination and its relationship to pathogenic tau species in animal models of tauopathy.

Our study demonstrates that SPD polyamination alters tau’s regulation of MT dynamics, promotes pathogenic conformations, and favors non-filamentous aggregation pathways. While these findings were derived from *in vitro* models, they are consistent with evidence of dysregulated polyamine pathways in tauopathy models and elevated SPD levels in AD brains [51,62,63,65,66]. Historically, *in vivo* studies have focused on the potential outcomes of cross-linking tau by TG and the modulation of polyamine metabolism pathways in the context of tau aggregation and pathology [52,54,56,58,97]. However, no studies have directly assessed the impacts of polyamination on tau biology. We found that SPD polyamination alters tau function by influencing MT polymerization and promoting the formation of pathogenic tau conformations. Our work suggests that SPD polyamination may direct tau toward non-filamentous pathways of aggregation that involve the adoption of known pathogenic tau conformations [95]. Collectively, these findings underscore the capacity of polyamination to significantly alter tau behavior, supporting further research into its role in normal physiology, tauopathies, and related neurodegenerative diseases. Future studies could explore the *in vivo* implications of SPD polyamination and its potential as a therapeutic target in tauopathies.

## Materials and methods

### Preparation of recombinant unmodified and SPD-modified tau proteins

Recombinant hT40 and hT39 tau proteins were prepared from a 4L bacterial culture as described previously [98], with the exception that BL21 bacteria (NEB, #C2527H) were used. The concentration of recombinant tau protein was determined using the BCA method (Thermo, # A53225). Next, polyamination reactions were performed *in vitro* by adapting the protocol described by Song and coworkers [99]. Briefly, 8 mM of SPD (Sigma, # S2626-1G) and 0.2 µM of TG enzyme (Sigma, # TS398) were added to 0.93 mg/ml of tau in 50 µl of reaction buffer (50 mM Tris HCl, pH 8, 10 mM CaCl_2_ and 5 mM DTT) followed by a 1 hour incubation at 37°C. Unmodified tau proteins were subjected to the same reaction conditions, but TG was excluded. Then, the TG enzyme was inactivated by heating at 70°C for 2 min. The TG-catalyzed reaction produces SPD-modified monomeric proteins as well as intra- and inter-molecular cross-linked proteins. To remove intermolecular cross-linked tau (i.e., high molecular weight species), SPD-modified tau was further purified by passing the sample through a 100 kDa MWCO Amicon filter (0.5 ml; Millipore, # UFC510096) (Supplementary Figure S1). Unmodified tau proteins were subjected to the same process. The flow-through (FL) was collected for further purification. Each concentrator membrane was washed six times with 400 µl buffer A (500 mM NaCl, 10 mM Tris, and 5 mM Imidazole pH 8). All the FL samples were pooled and subsequently buffer exchanged into Buffer A using a HiPrep 26/10 desalting column (Cytiva, #17508701) and fast protein liquid chromatography (FPLC). The desalting column was equilibrated with 5 column volumes (CVs) of buffer A, then protein samples were run over the column in 5 CVs of buffer A at a flow rate of 5 ml/min, and fractions containing tau were collected. Finally, to purify the tau proteins (6× His-tagged) from other proteins in the polyamination reaction (e.g., TG) and free SPD, heavy metal affinity chromatography was used using a 5 ml HiTrap Talon crude column (Cytiva, #28953767). The HiTrap Talon column was equilibrated in 5 CVs of buffer A, the proteins were run over the column, the column was washed with 5 CVs of buffer A, and then tau proteins were eluted in 10 CVs of buffer B (100 mM imidazole in buffer A, pH 8, supplemented with 200 µM PMSF) at a flow rate of 3 ml/min in 5 ml fractions. Fractions containing highly purified monomeric tau (determined using SDS-PAGE) were pooled and concentrated to 2–4 mg/ml using an Amicon^®^ Ultra Centrifugal Filter, 30 kDa MWCO (Millipore, # UFC903008), then DTT was added (final concentration of 1 mM). The unmodified hT40 (hT40), SPD-hT40, unmodified hT39 (hT39), and SPD-hT39 samples were then aliquoted and frozen at −80°C. The final concentration of recombinant tau proteins was determined using the SDS-Lowry method as described previously [98].

### Western blot validation of recombinant tau proteins

To confirm the polyamination status of recombinant hT40, SPD-hT40, hT39, and SPD-hT39 proteins, 0.5 μg of protein was loaded on 4–20% TGX gels (Biorad, # 5671095) and run at 250 V for 32 min, followed by transfer onto nitrocellulose membranes (LI-COR Biosciences, # 926–31092) using the BioRad wet transfer system at 400 mAmp for 50 min. The membranes were then blocked with 2% non-fat dry milk (NFDM) in 1X tris-buffered saline (TBS) for 1 hour at room temperature, followed by incubation in primary antibodies overnight at 4°C. Primary antibodies were Tau5 (Nicholas M. Kanaan at Michigan State University, RRID: AB_2721194) [30,100,101] at 1:100,000 in 2% NFDM and anti-spermine (Abcam, # ab26975, RRID: AB_470871, cross-reacts 100% with SPD) at 1:2,000 in 2% NFDM. The following day, the blot was washed three times (5 min each) with TBS supplemented with 0.1% Tween 20 (TBS-T). Then, secondary antibodies in 2% NFDM were added for 1 hour at room temperature. Secondary antibodies were goat anti-mouse IgG1 680 (LI-COR Biosciences, # 926–68050, RRID: AB_2783642) at 1:20,000 and goat anti-rabbit IgG 800 (LI-COR Biosciences, # 926–32211, RRID: AB_621843) at 1:20,000. The blot was then washed three times (5 min each) with TBS-T. LI-COR Odyssey classic imager and Image Studio Lite Ver 5.2 were used to visualize the blots.

### Preparation of recombinant tau protein for tandem MS

The hT40, SPD-hT40, hT39, and SPD-hT39 proteins were digested using a combination of trypsin (Promega, #V5280) and rLysC (Promega, #V167A). First, each protein (3 µg) was subjected to five rounds of buffer exchange with 25 mM ammonium bicarbonate (AmBic) pH 8 using a 0.5 ml 3 kDa MWCO Amicon centrifugal filter (15,000×g for 10 min; Millipore, # UFC500396). Then, the tau proteins were retrieved by inverting the filter into a recovery tube, centrifugation at 15,000×g for 2 min, and then vacuum drying using Vacufuge. The dried pellets were reconstituted in 50 µl of digestion buffer (12.5 mM AmBic, pH 8 + 50% acetonitrile (ACN)] and incubated at 37°C for 90 min with rLysC (150 ng of enzyme per 3 μg of recombinant protein). Then, trypsin was added (300 ng of enzyme per 3 μg of recombinant protein) and incubated at 37°C for 16–18 hours. The following day, digested protein samples were vacuum dried and stored at −20°C until running on MS.

### Tandem MS of recombinant tau proteins

We used a Thermo Scientific Ultimate 3000 RSLCnano System coupled with nanoscale liquid chromatography. Desalting of digested peptides was conducted in-line using a 3 μm diameter bead, C18 Acclaim PepMap trap column (75 μm × 20 mm) with 2% ACN, 0.1% formic acid (FA) for 5 min at a flow rate of 2 μl/min at 40°C. The trap column was then brought in line with a 2 μm diameter bead, C18 EASY-Spray column (75 μm×250 mm) for analytical separation over 128 min with a flow rate of 350 nl/min at 40°C. The mobile phase included two buffers: 0.1% FA (Buffer A) and 0.1% FA in ACN (Buffer B), and a gradient was used for separation as follows: 12.5 min desalting, 95 min 4–40% B, 2 min 40–65% B, 3 min 65–95% B, 11 min 95% B, 1 min 95–4% B, and 3 min 4% B. We injected 1 μg of each sample for analysis. Top 20 data-dependent MS analysis was performed with a Q Exactive HF-X Hybrid Quadrupole-Orbitrap Mass Spectrometer. MS1 resolution was 60K at 200 m/z with a maximum injection time of 45 ms, AGC target of 3e6, and scan range of 300–1500 m/z. MS2 resolution was 30K at 200 m/z, with a maximum injection time of 54 ms, AGC target of 1e5, and isolation range of 1.3 m/z. High-energy collision dissociation normalized collision energy was 28. Only ions with charge states from +2 to +6 were selected for fragmentation, and dynamic exclusion was set to 30 s. The electrospray voltage was 1.9 kV at a 2.0 mm tip-to-inlet distance. The ion capillary temperature was 280°C, and the RF level was 55.0. All other parameters were set as default.

### MS data analysis to determine SPD modification sites

RAW data files were analyzed with the MetaMorpheus software version 1.0.1 developed by the Smith laboratory [102]. For hT40 proteins, the following databases were downloaded from Uniprot (November 2021) and used for analysis: Escherichia coli (strain K12) (UP000000625), trypsin (Q29463), Lys-C (Q02SZ7), and full-length tau sequence (2N4R isoform, P10636-8). The same files were used to analyze the hT39 proteins using the 2N3R tau isoform sequence (P10636-5) instead of the 2N4R sequence. Mass shifts corresponding to the non-acetylated SPD were used to search for modifications: +128.1313485 for SPD [103,104]. In addition, the fragmentation pattern of SPD was determined by running SPD alone on MS. Mass-to-charge ratios (m/z) corresponding to diagnostic ions (DIs) were identified: 54.048, 57.059, 71.075, 111.109, and 128.132. The search parameters for the SPD modification included both mass shift and the identified diagnostic ions.

The analysis sequence included mass calibration, global post-translational modification discovery (G-PTM-D) [105], and a classic search. Mass calibration was conducted using the following criteria: protease = trypsin; maximum missed cleavages = 2; minimum peptide length = 7; maximum peptide length = unspecified; initiator methionine behavior = variable; variable modifications = oxidation on M; max mods per peptide = 2; max modification isoforms = 1024; precursor mass tolerance = ± 15.0 ppm; product mass tolerance = ± 25.0 ppm. The criteria utilized for G-PTM-D were protease = trypsin; maximum missed cleavages = 2; minimum peptide length = 7; maximum peptide length = unspecified; initiator methionine behavior = variable; max modification isoforms = 1024; variable modifications = oxidation on M; G-PTM-D modifications count = 3; precursor mass tolerance(s) = ± 5.0 ppm around 0 ,128.131348525 Da; product mass tolerance = ± 20.0 ppm. Finally, a classic search was conducted using the following criteria: protease = trypsin; search for truncated proteins and proteolysis products = false; maximum missed cleavages = 2; minimum peptide length = 7; maximum peptide length = unspecified; initiator methionine behavior = variable; variable modifications = oxidation on M; max mods per peptide = 2; max modification isoforms = 1024; precursor mass tolerance = ± 5.0 ppm; product mass tolerance = ± 20.0 ppm; report peptide spectral match (PSM) ambiguity = true. SPD polyamination site of tau detected at a false discovery rate of 1% is reported (Supplementary Table S1). online supplementary table 2Supplementary Table S2 demonstrates all quantified tau peptides in unmodified vs. SPD-modified tau samples. Supplementary Table S3 shows the quantified peaks of tau with their corresponding peptide masses, theoretical and observed m/z, retention time, and PSMs. MetaDraw version 1.0.5 was utilized to review the PSMs of modified and unmodified tau peptides (samples of these peptides are included in Supplementary Figures S2 and S3). Full proteomics data sets and .RAW files from MS are available in a public repository (https://doi.org/10.5061/dryad.59zw3r2jp).

### Tubulin polymerization assay

Tubulin polymerization in the presence of hT40, SPD-hT40, hT39, or SPD-hT39 proteins was assessed using the Tubulin Polymerization Assay Kit (Cytoskeleton, #BK011P). Kit reagents were reconstituted and stored as indicated in the manual. The assay began by incubating the 96-well plate provided with the kit at 37°C for 10 min (Synergy H1 Hybrid Multi-Mode Reader and Gen5 software v3.11, BioTek). The tau proteins were prepared as 10 μM stocks in general tubulin buffer (80 mM PIPES pH 6.9, 2 mM MgCl_2_, and 0.5 mM EGTA) and left at room temperature. Then, tubulin master mix was prepared and kept on ice using the recipe for enhancer detection: 355 μl of Buffer 1, 4.4 μl of 100 mM GTP, and 85 μl of tubulin 10 mg/ml. Once the 10 min incubation of the 96-well plate was complete, recombinant tau proteins (10 μM stocks) were added (5 μl/well) to the plate and incubated at 37°C for 1 min. Then, the tubulin master mix was added (50 μl/well) yielding a final tau concentration of 1 μM. Fluorescence signal was measured for 1 hour to monitor tubulin polymerization using the kinetic mode at excitation and emission wavelengths of 360 nm and 450 nm, respectively. Each tau protein sample was loaded in duplicate (technical replicates), and the experiment was performed four times by making four independent sets of samples (biological replicates). Background levels of blank (general tubulin buffer only) were subtracted from the fluorescence readings before further analyzing the data. Nonlinear regression using ‘specific binding with Hill slope’ was used to fit the tubulin polymerization data in GraphPad Prism v10.2.1 and calculate the steady state equilibrium (*V*_max_) along with the time to half-maximal polymerization (*Kd*).

### MT-binding assay

The ability of hT40, SPD-hT40, hT39, and SPD-hT39 proteins to bind pre-formed MTs was assessed using the Microtubule Binding Protein Spin-Down Assay Biochem Kit (Cytoskeleton, # BK029). Kit reagents were reconstituted as indicated in the manual. Tubulin aliquots (20 μl) were thawed, supplemented with cushion buffer (2 μl; 80 mM PIPES pH 7.0, 1 mM MgCl_2_, 1 mM EGTA, and 60% sucrose), and incubated at 35°C for 40 min. General tubulin buffer (200 μl; 80 mM PIPES pH 7.0, 1 mM MgCl_2_, and 1 mM EGTA) supplemented with paclitaxel at 20 μM was added to the polymerized tubulin. MT-binding reactions were set up at room temperature including tubulin (20 μl, 2 µM tubulin dimer), recombinant tau proteins (0.5 μM), and general tubulin buffer to a final volume of 50 μl. After 30 min of incubation at room temperature, binding reactions were loaded on 100 μl of 60% sucrose cushion buffer in 0.2 ml polycarbonate tubes (Thermo Scientific, # 45233) followed by centrifugation at 100,000×g for 40 min. For all centrifugation steps, an S100-AT3 Fixed Angle Rotor (Thermo Scientific, # 45585) and a Sorvall™ MTX 150 Micro-Ultracentrifuge (Thermo Scientific, # 46960) were used. Then, 30 μl of the supernatant was carefully removed to avoid disturbing the MT pellet. Laemmli buffer was added to the supernatant immediately (6 μl of 6X Laemmli buffer). The rest of the supernatant was discarded. The MT pellet was washed with general tubulin buffer (100 μl) supplemented with 20 μM paclitaxel. The MT pellet was subjected to centrifugation at 100,000×g for an additional 20 min. The previous washing step was repeated once, followed by careful removal of the supernatant. The final pellet was resuspended in 60 μl of 1X Laemmli buffer. The assay was conducted four times by making four independent sets of samples (biological replicates).

For each biological replicate, the supernatant and pellet (15 μl each) fractions were subjected to SDS-PAGE. The hT40, SPD-hT40, hT39, and SPD-hT39 samples were loaded alongside Precision Plus Protein™ All Blue Prestained Protein Standard (Biorad, #1610373) on two separate 4–20% Novex tris-glycine gels (Invitrogen, # WXP42020BOX) and run at 225 V for 45 min. After SDS-PAGE, the two gels were cut between the 250 and 37 kDa marker bands and added to the same transfer cassette for transfer onto a nitrocellulose membrane. This step allows for the quantification of all tau bands on the same blot to minimize the variability in the blotting procedure across samples. The rest of the blotting procedure was performed as described above. Primary antibodies used were the Tau5 antibody at 1:100,000 and the tubulin 5H1 antibody at 1:5,000 (Nicholas M. Kanaan at Michigan State University, RRID: AB_2832941) [106]. Secondary antibodies used were goat anti-mouse IgG1 680 and goat anti-mouse IgM 800 (LI-COR Biosciences, # 926–32280, RRID: AB_2814919) at 1:20,000 each. LI-COR Odyssey classic imager and Image Studio Lite Ver 5.2 were used to visualize fluorescent signals of the antibody probes. Tau bands were quantified in all supernatant and pellet fractions, followed by calculating the percentage of tau in pellet relative to total tau using the following equation:


$$\%\;Tau\;in\;Pellet\;=\;\frac{Tau\;in\;Pellet}{\left(Tau\;in\;Pellet\;+\;Tau\;in\;Sup\right)}\;\times \;100$$

### *In vitro* recombinant tau aggregation reaction

Aggregation of different tau proteins was induced by arachidonic acid (ARA; Cayman Chemical, # 90010) in 200 μl reactions as described previously [107,108]. Briefly, 2 μM of tau (M. Wt. = 43426 Da for hT39; M. Wt. = 46673 Da for hT40) was induced to aggregate *in vitro* by incubation in tau aggregation buffer (10 mM sodium HEPES, 0.1 mM EDTA, 200 mM NaCl, 5 mM DTT, pH 7.6) with (aggregated) or without (unaggregated; ethanol vehicle was used) 75 μM ARA. ARA stocks were prepared in 100% ethanol at 2 mM immediately before use and kept on ice. The ARA was added as the final component in the reaction sample, and then the samples were gently mixed by minimally shaking the tube/cuvette. Aggregation reactions were incubated at room temperature and monitored using laser light scattering for 6 hours (see below). Unaggregated tau reactions were aliquoted and frozen immediately after the addition of 100% ethanol.

### Right-angle LLS

The right-angle LLS assay measures tau aggregation kinetics over time [107,109]. LLS was measured using a class IIb laser with a wavelength of 475 nm and maximum power of 20 mW (B & W INC., model # BWI-475–20-E) and a digital camera (Thor Labs, model # DCC1240M). Images were acquired using uc480 Viewer version 4.2 with the pixel clock set at 11 MHz. Images were acquired for hT40 and SPD-hT40 samples at a frame rate of 2 fps and exposure of 250 ms, while a frame rate of 1 fps and exposure time of 300 ms was used for hT39 and SPD-hT39 samples. Polymerization reactions of tau (prior to ARA addition) were transferred into glass cuvettes with a path length of 5 mm (Starna Cells, #3 G-5), and LLS was measured at time zero before the addition of ARA to obtain baseline measurements. After the addition of ARA and gently mixing the samples, images were serially acquired at 1, 2, 3, 4, 5, 10, 15, 20, 25, 30, 35, 40, 45, 50, 55, 60, 75, 90, 105, 120, 150, 180, 240, 300, and 360 min. Each experiment was conducted four independent times. Image analysis was performed with Adobe Photoshop CS6 (Adobe Systems INC.) using the marquee tool. The region of interest used for densitometry measurements was set to 150 pixels x 15 pixels and adjusted to the center of the glass cuvette within the band of scattered light. Pixel intensity was recorded using the histogram feature. Scattered light intensity (intensity) measurements during the 6 hours were fitted using the nonlinear Gompertz function [24,110-112]:


$$y=a{\text{e}}^{\text{-}{\text{e}}^{\text{-}\frac{\text{t-}{\text{t}}_{\text{i}}}{\text{b}}}}$$

The Gompertz equation describes polymerization kinetics using the following three parameters [111,112]: *y* = light scattering at a given time *t; t_i_* is the inflection point at maximal light scattering; *a* = the maximum light scattering at equilibrium; *b = 1/k_app_*, where *k_app_* is the proportional growth rate of the filament population in units of time^–1^. Lag times, defined as the time before detectable polymerization occurs, were calculated as *t_i_ – b*.

### ThS fluorescence

At the end of the tau aggregation reaction with ARA (described above), β-sheet-containing aggregates were quantified using a ThS assay as previously described [24,107]. Immediately before starting the assay, a 0.0175% ThS solution was prepared in water, filtered through a 0.22 µM membrane, and protected from light. Then, 150 μl of each tau sample was mixed with 6 μl of ThS solution and incubated for 5 min at room temperature. The samples (150 µl/well) were then loaded into a black-walled 96-well plate (Costar, # 3915), and fluorescence was immediately read using the Promega Glomax multi-detection system at 490 nm excitation and 510–570 nm emission wavelengths. Control buffers for unaggregated and aggregated reactions were included to obtain background measurements, then their absorbance values were subtracted from the tau sample values.

### Quantitative TEM measurements

Transmission electron microscopy (TEM) allows for the visualization and measurement of tau aggregates [107]. First, 1 ml of 2% uranyl acetate (UA; Electron Microscopy Sciences, # 22400) solution was freshly prepared in deionized water (DIW) at room temperature. The solution was filter sterilized using a 0.22 μM membrane (Fisher Scientific, # 13–1001-06). hT40, SPD-hT40, hT39, and SPD-hT39 samples (10 μl) were fixed with 2% glutaraldehyde (Electron Microscopy Sciences, # 16100) at room temperature for 10 min. Then, each sample (5 μl) was absorbed onto a formvar-coated copper grid (Electron Microscopy Sciences, #FCF300-CU) for 1 minute, followed by one rapid rinse in DI water and another rapid rinse in UA solution. Finally, the grids were incubated with UA solution for 1 min at room temperature, then the solution was wicked away, and grids were left to dry for at least 1.5 hours before imaging. Grids were prepared of unaggregated and aggregated tau (four biological replicates of each were prepared independently) and imaged using a JEOL JEM-1400 Plus electron microscope at 80 kV. Electron micrographs were captured with an AMT XR81 digital camera and AMT software version 602.6.

For each grid, three images were captured at 5,000× magnification for quantitative TEM. Electron micrographs were processed using ImageJ v1.54 using a method like that described by Tiernan and coworkers [107]. First, the scale bar was calibrated to 373 pixels/800 nm. Then, images were smoothed three times to allow for the automatic thresholding to capture the visible tau aggregates. Finally, particle count, size (nm^2^), and the total area occupied by tau aggregates (% area) measurements were collected using the ‘Analyze Particles’ command (size = 0-infinity; circularity = 0–1). The data were prepared for analysis and graphing using GraphPad Prism v10.2.1. Frequency distribution plots using 700 nm^2^-wide and 50 nm^2^-wide bins for hT40 and hT39, respectively, were created. The sum of particles in three images/independent replicates was calculated and counted as *n* = 1 independent replicate, with a total of four independent replicates. The following populations of hT40 were plotted according to criteria similar to those reported by Tiernan et al. [107]: < 700 nm^2^ for globular aggregates only; 700–2100 nm^2^ for globular aggregates > 700 nm^2^ along with short filaments; 2100–5000 nm^2^ for short filaments only; > 5000 nm^2^ for long filaments only. Aggregates of hT39 were globular in nature and split into sizes smaller or larger than 450 nm^2^.

### Western blot for unaggregated and aggregated reactions to measure stable multimers

The hT40, SPD-hT40, hT39, and SPD-hT39 samples were prepared for SDS-PAGE by diluting to 0.5 μM in 1× Laemmli sample buffer. Samples were boiled at 95°C for 5 min, followed by vortexing and a quick spin down. Samples (0.5 µg/lane) were loaded on a 20-well 4–20% Novex tris-glycine gel and run as described above. Proteins were transferred to a nitrocellulose membrane using the BioRad wet transfer system (as described above). The blot was blocked with 2% NFDM for 1 hour at room temperature, followed by incubation with Tau5 antibody (1:100,000 in 2% NFDM) overnight at 4°C. The following day, the membrane was washed three times with TBS-T, 5 min each. Then, membranes were incubated with goat anti-mouse IgG1 680 secondary antibody (1:20,000 in 2% NFDM) at room temperature for 1 hour. Membranes were washed with TBS-T three times, 5 min each, then imaged using LI-COR Odyssey classic imager and Image Studio Lite Ver 5.2. Tau bands corresponding to monomeric tau (monomer band) and higher molecular weight multimers (HMW bands) were quantified.

### Sandwich enzyme-linked immunosorbent assay (sELISA)

To detect pathogenic tau conformations using conformation-dependent antibodies (i.e., TOC1, TOMA1, TNT2, and Alz50), tau samples must be kept under native conditions [29,67,73,98,113]. Therefore, sELISA assays were used to measure pathogenic tau conformations in 4 independent sets of samples (biological replicates). All steps were performed at room temperature. The following capture antibodies were used: TOC1 (Kanaan Lab, RRID: AB_2832939) [21,67], TOMA1 (Millipore, #MABN819) [113], TNT2 (Kanaan Lab, RRID: AB_2736931) [73,74], Alz50 (P. Davies Albert Einstein College of Medicine, New York, USA, RRID: AB_2313937) [29,30,75], or Tau13 (Kanaan Lab, RRID: AB_2721193) to measure total tau [114]. Capture antibodies were diluted to 2 ng/μl in borate saline buffer (100 mM borate acid, 25 mM sodium borate, 75 mM NaCl, 0.25 mM thimerosal). Then, high-binding capacity 96-well plates (Corning, #3590) were coated with the capture antibodies (50 μl/well) for 1 hour. Wells were washed two times with ELISA wash buffer (200 μl/well; 100 mM borate acid, 25 mM sodium borate, 75 mM NaCl, 0.25 mM thimerosal, 0.4% (w/v) bovine serum albumin, and 0.05% (v/v) Tween-20), then blocked with 5% NFDM in ELISA wash buffer (200 μl/well) for 1 hour. Two washes with ELISA wash buffer were performed, followed by the addition of unaggregated and aggregated tau samples for 1.5 hours. The hT40 and SPD-hT40 samples were prepared in TBS at the following concentrations (50 μl /well): 2.5 nM for Tau13; 5 nM for TNT2; 20 nM for TOC1 and Alz50; 150 nM for TOMA1. The hT39 and SPD-hT39 samples were prepared in TBS at the following concentrations (50 μl/well): 2.5 nM for Tau13; 40 nM for Alz50; 100 nM for TNT2; 150 nM for TOC1 and TOMA1. Then, wells were washed four times with ELISA wash buffer (200 μl/well). The detection antibody R1 (Kanaan Lab, RRID: AB_2832929) [115] was diluted at 1:10,000 in 5% NFDM and added to the wells (50 μl/well) for 1.5 hours in all assays. Then, wells were washed four times with ELISA wash buffer and incubated with Goat Anti-Rabbit IgG Antibody (H + L), Peroxidase secondary antibody (Vector Laboratories, #PI-1000–1, RRID: AB_2916034) at 1:5,000 in 5% NFDM for 1 hour (50 μl/well). After four final washes with ELISA wash buffer, the peroxidase reaction was developed using 3,3′,5,5′-Tetramethylbenzidine (TMB; 50 μl/well; Sigma, # T0440). The peroxidase reaction was stopped with 4% sulfuric acid, followed by reading the absorbance at 450 nm using SpectraMax Plus 384 microplate reader (Molecular Devices). The absorbance values (A) were further processed in GraphPad Prism v10.2.1 to calculate percentage light absorbed (% Light Abs) using the following equation:


$$\%\text{ Light Abs}=100-\left(100*10^{-\text{A}}\right)$$

### Statistics

Statistical analyses were performed using GraphPad Prism v10.2.1. Two-tailed *t*-test was used to analyze the following results: *V*_max_ and *Kd* of tubulin polymerization assay; lag time, *k*_app_, and max LLS; % area from quantitative TEM for hT40. Mann–Whitney test was used (violation of normality) for % area from quantitative EM for hT39. Two-way analysis of variance (ANOVA) followed by the post-hoc Holm-Sidak with all possible comparisons was used to analyze the following results: % Tau in pellet of MT-binding assay; ThS signal; Monomer and HMW bands for stable multimers; % Light Abs of sandwich ELISA assays. Differences in outcomes were deemed statistically significant at *P*≤0.05.

## Supplementary material

Online supplementary figures

Online supplementary table 1

Online supplementary table 2

Online supplementary table 3

## Data Availability

All the data are provided in this manuscript, the supplemental information and/or on Dryad (https://doi.org/10.5061/dryad.59zw3r2jp). Any additional information is available upon reasonable request to Dr. Nicholas Kanaan (corresponding author) at nkanaan@msu.edu.

## Abbreviations

AD: Alzheimer’s disease
MT: microtubule
PTMs: post-translational modifications
SPD: spermidine
TG: transglutaminase
ThS: thioflavin S
