# Supplementary material for: Polyamination with spermidine enhances pathogenic tau conformations while reducing filamentous aggregate formation in vitro
Source: Biochem J. 2025 Jun 17;482(12):877–99. doi: 10.1042/BCJ20253079 (PMC12235667; doi:10.1042/BCJ20253079)
Supplement: Online supplementary figures [file bcj-482-12-BCJ20253079-supp1.docx]

**­­­Supplementary Materials:**

**Polyamination with spermidine enhances pathogenic tau conformations while reducing filamentous aggregate formation *in vitro***

Mohammed M. Alhadidy, Rebecca L. Mueller, Jared Lamp, and Nicholas M. Kanaan

**Supplementary Figure 1.**


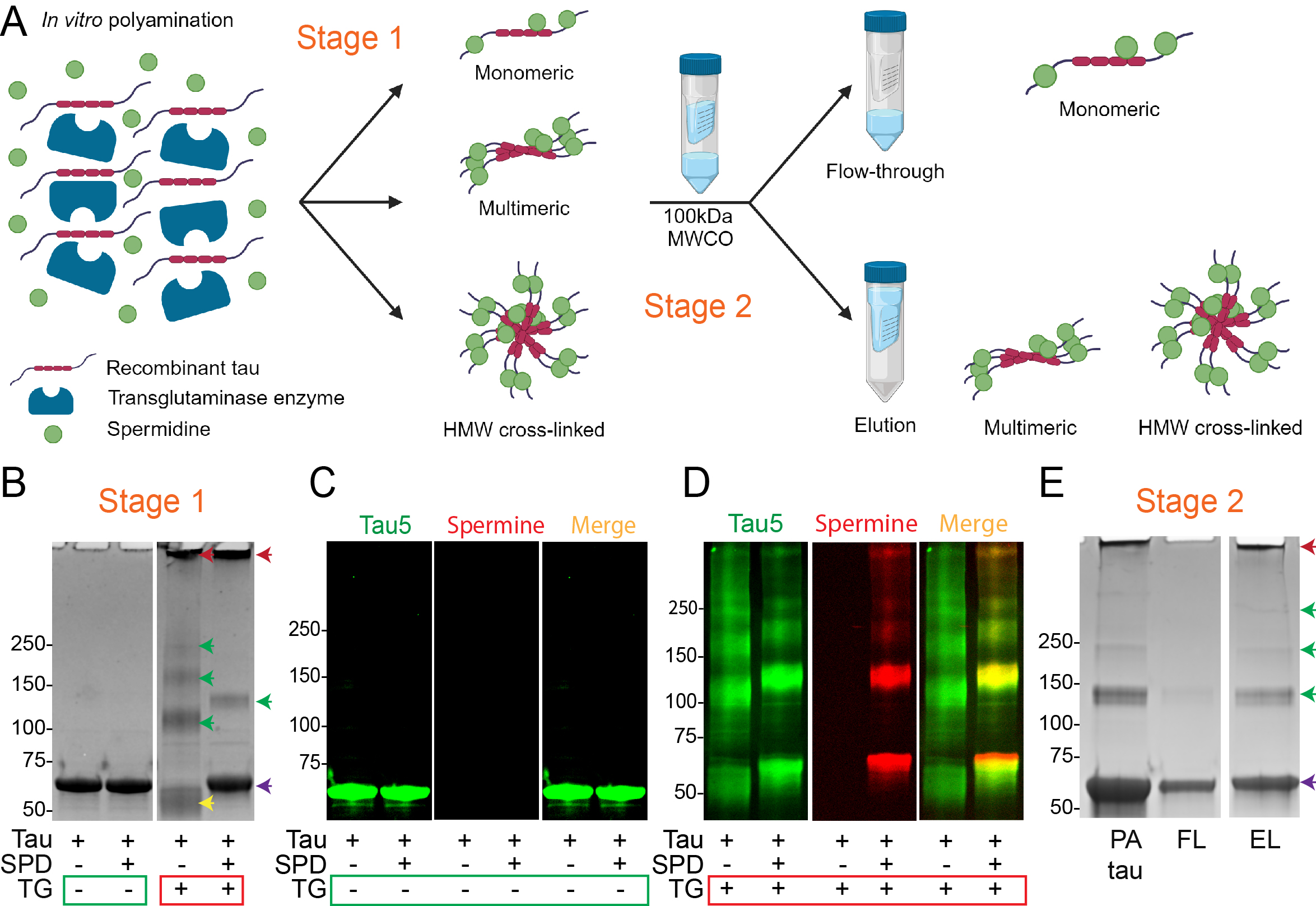


**Figure S1. Production and purification strategy of spermidine (SPD)-modified tau using 2N4R tau isoform (hT40) as an example.**

A, *in vitro* polyamination reaction with SPD is set up in the presence of the transglutaminase (TG) enzyme. The polyamination reaction (stage 1) produces a mixture of tau species that includes polyaminated monomeric, multimeric, and high molecular weight (HMW) crosslinked tau. Passing the heterogeneous mixture of polyaminated tau products through a membrane with 100 kDa molecular weight cutoff (MWCO) (stage 2) separates the polyaminated monomeric (flow-through) from multimeric and crosslinked tau species (elution). B-D, mixing tau and SPD in the absence of TG does not produce SPD polyaminated/crosslinked tau (stage 1). A Coomassie stained gel (B) shows that in the absence of SPD, TG gives rise to a mixture of crosslinked products that includes intraprotein crosslinked (yellow arrowhead), multimeric (green arrowheads), and HMW interprotein crosslinked (red arrowhead) tau species. Adding SPD to the reaction dramatically decreases the intraprotein crosslinked tau species while producing SPD polyaminated monomeric tau (purple arrowhead). Immunoblot of samples (C) shows that SPD is not incorporated into tau proteins in the absence of TG enzyme. In contrast, the presence of TG alone (D) produces a mixed population of intraprotein and interprotein crosslinked tau species. When both TG and SPD are present, polyaminated monomeric, multimeric, and HMW crosslinked tau species are produced. E, the polyaminated tau (PA tau) was passed through a membrane using a 100 kDa MWCO filter (stage 2). The flow-through (FL) contains mainly monomeric tau species (purple arrowhead), while the HMW tau species were mostly separated into the elution (EL). Therefore, PA tau in the FL was used to conduct the experiments described in this work. The same strategy was used to purify a polyaminated version of the longest 3R tau isoform–hT39.

**Supplementary Figure 2.**


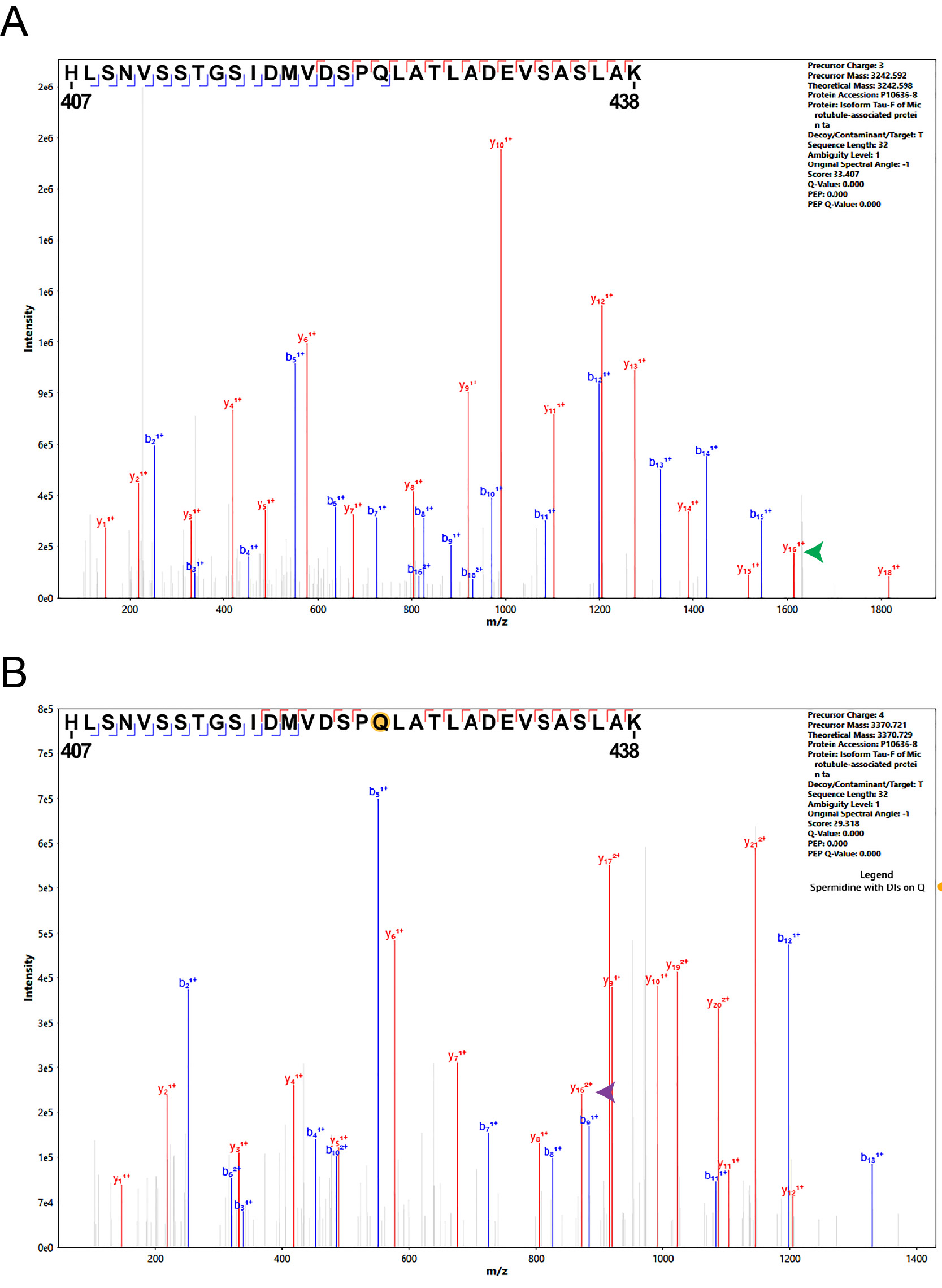


**Figure S2. Sample mass spectra of tau peptides showing spermidine (SPD) modification at glutamine 424 (Q424) of 2N4R tau isoform (hT40).**

A, mass spectrum of peptide spanning amino acids 407-438 in unmodified hT40, showing all fragmented y-ions with no mass change corresponding to polyamination with SPD, such as y_16_^1+^ m/z = 1613.88 equivalent to a mass of 1612.88 Da (green arrowhead). B, mass spectrum of peptide spanning amino acids 407-438 in SPD-modified hT40 (SPD-hT40), with fragmented y-ions from y_16_-y_20_ carrying a double positive charge and a mass shift corresponding to polyamination with SPD, such as y_16_^2+^ m/z = 871.51 equivalent to a mass of 1741.02 Da (purple arrowhead). There was also a reduction in retention time of SPD-modified peptide relative to unmodified peptide 407-438.

**Supplementary Figure 3.**


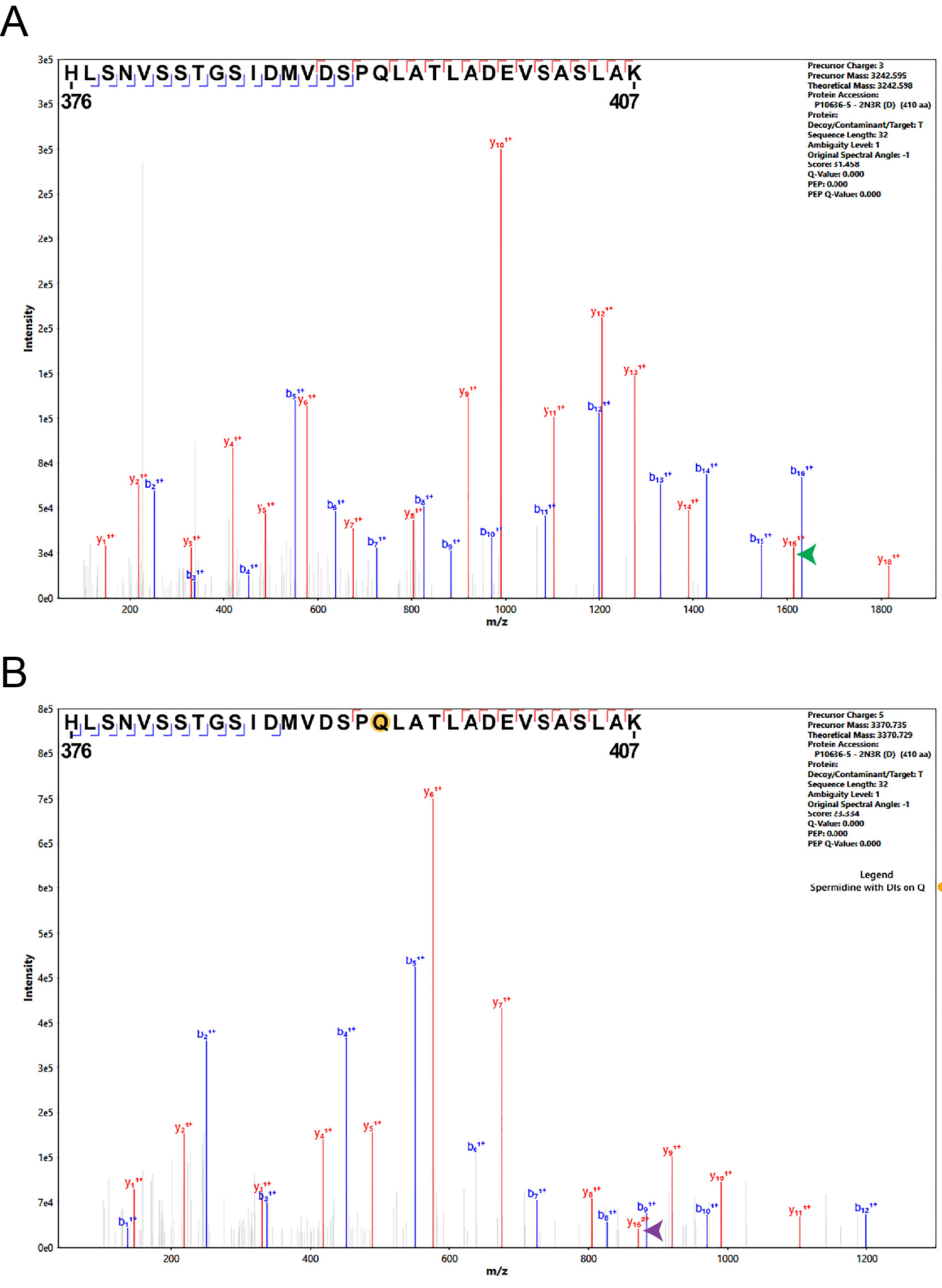


**Figure S3. Sample mass spectra of tau peptides showing spermidine (SPD) modification at glutamine 393 Q393 of 2N3R tau isoform (hT39).**

A, mass spectrum of peptide spanning amino acids 376-407 (corresponds to 407-438 of 2N4R tau isoform) in unmodified hT39. All fragmented y-ions showed no change in mass corresponding to polyamination with SPD (e.g., y_16_^1+^ m/z = 1613.87 equivalent to a mass of 1612.87 Da; green arrowhead). B, mass spectrum of peptide spanning amino acids 376-407 in SPD-modified hT39. Fragmented y_16_^2+^ carries a double positive charge with a mass shift corresponding to polyamination with SPD (i.e., y_16_^2+^ m/z = 871.51 equivalent to a mass of 1741.02 Da; purple arrowhead). There was also a reduction in retention time of SPD-modified peptide relative to unmodified peptide 376-407.
